# Supplementary material for: A Vaspin–HSPA1L complex protects proximal tubular cells from organelle stress in diabetic kidney disease
Source: Commun Biol. 2021 Mar 19;4:373. doi: 10.1038/s42003-021-01902-y (PMC7979793; doi:10.1038/s42003-021-01902-y)

Supplementary Information

A Vaspin - HSPA1L complex protects proximal tubular cells from organelle stress in diabetic kidney disease

Atsuko Nakatsuka, Satoshi Yamaguchi, Jun Eguchi, Shigeru Kakuta, Yoichiro Iwakura,
Hitoshi Sugiyama and Jun Wada

Supplementary Methods

Gene expression of Hspa1l in C57BL/6J mouse kidney

Total RNA was isolated from kidney tissue of C57BL/6J mouse and cDNA was synthesized. Then, cDNA was subjected to PCR using primers: sense 5'-TCACGGTGCCAGCCTATTTC-3' and antisense 5'-CGTGGGCTCATTGATTATTCTCA-3'. *Hspa1l* gene was detected as a 101 bp band in 4% agarose gel.

Supplementary Table 1

Primer	
3xFLAG-F	5'- GGGGGGGGATCCATGGACTACAAAGACCAT-3'
3xFLAG-R	5'- GGGGGGGGATCCCTTGTCATCGTCATCCTT-3'
Flag-*EcoRI*-hVaspin-F	5'-GGGGGGGGAATTCACTAAAGCCGAGCTTCTCACC-3'
OL64-pTNT-AS	5'-GGGGGGTCTAGATTATTTTCCAATAGGGTTAA-3'
Flag-*HindIII*-GRP78 -S	5'-GGGGGGAAGCTTTGAGGAGGAGGACAAGAAG-3'
GRP78-AS1-RE	5'-GGGGGGGCGGCCGCCTACAACTCATCTTTTTC-3'

Supplementary Figure Legends

Supplementary Figure 1

Vacuolation of proximal tubules in Vaspin transgenic (Tg), wild type (WT) and Vaspin-/- mice under high fat-high sucrose (HFHS) diet at 30 weeks of age.

a. Immunohistochemical staining of aquaporin 1 (AQP1) in kidney tissues of Vaspin Tg, WT and Vaspin-/- mice. Prominent vacuolation is observed in AQP-positive proximal tubules in Vaspin-/- and WT mice under HFHS. P; proximal tubules, D; distal tubules, G; glomerulus. Bar=100 µm. **b**. Toluidine blue-positive vacuoles are enlarged and increased in Vaspin-/- mice under HFHS diet, while they are ameliorated in Vaspin Tg mice. Bar=100 µm. **c.** Electron micrographs of enlarged lysosomes in tubules from Vaspin-/- mice fed with HFHS. lysosomes are indicated by arrows. **d**. Vacuolation score. HFHS diet induced vacuolation in

Vaspin Tg, WT and Vaspin-/- mice compared with STD. In Vaspin Tg mice, vacuolation is inhibited compared to WT and Vaspin-/- mice under HFHS diet. **e**. Dilatation score. There are no statistical differences among Vaspin Tg, WT, and Vaspin-/- mice under STD or HFHS diet. N=4-17. Data are shown as mean ± S.D. Bonferroni method is used for multiple-comparison corrections. **f**. Immunohistochemical staining of p62. Bar = 50 μm. **g**. Percent of p62 positive area. N=3-5 independent mice, one-way ANOVA with Tukey-Kramer method is used. Data are shown as mean ± S.D. **h**. Glomerulus in PAS staining. Bar = 50 μm. **i.** Mesangial matrix index. N=4-8, one-way ANOVA with Tukey-Kramer method is used. **j.** Urinary albumin in WT under STD, Vaspin Tg, WT and Vaspin-/- under HFHS diet., One-way ANOVA with Tukey-Kramer method is used. Data are presented as mean ± S.D., *p<0.05, **p<0.01. Histochemical analyses were performed twice.

Supplementary Figure 2

ER stress induced-lysosomal enlargement and p62 accumulation.

HK2 cells were cultured with DMSO or 1 μg/ml tunicamycin (TM) or 1 μM thapsigargin (TG) for 12 hr. Recombinant human vaspin (rhVaspin) was co-administrated at 100 ng/ml for 12 hr. **a** Lysosome stained with Lysotracker (red) and Lamp1 (green). Bar=25 μm. **b**. Western blot analyses of HK2 cells cultured with 1 μg/ml TM, 1 μM TG, and 100 ng/ml rhVaspin for 24 hr. **c**. Average size of particle in Lysotracker Red staining. **d**. Quantification of western blots in panel. N=3-6. Statistical analyses are performed in following groups; group 1 (DMSO, TM, DMSO+rhVaspin, and TM+rhVapsin) and group 2 (DMSO, TG, DMSO+rhVaspin, and TG+rhVaspin). The identical data of DMSO or DMSO+rhVaspin are used both in groups 1 and 2, because the analyzed samples were run on the same membrane in panel **c**. Phospho-eIF2α/eIF2α ratio in both TM and TG treatment experiment and CHOP/αTubulin ratio in TG treatment experiment are analyzed by Bonferroni method. The rest of blots are analyzed by one-way ANOVA with Tukey-Kramer method. **e**. TUNEL staining of HK2 cells cultured with 0 or 1 μg/ml TM for 24 hr. Bar=100 μm. Apoptotic cells are indicated by white arrows. **f.**

Percentage of HK2 cells with TUNEL positive apoptotic cells per field from 11-24 fields. One-way ANOVA with Tukey-Kramer method is used. All data are presented as mean ± S.E, *p < 0.05, **p < 0.01. Western blots and staining of the cells were performed twice.

Supplementary Figure 3

Palmitate-induced lysosomal membrane permeabilization in HK2 cells demonstrated by galectin puncta assay.

HK2 cells expressing pGFP-galectin-3 were cultured with 500 μM palmitate (PA) or 0 μM (CTRL) for 12 hr. rhVaspin was co-administrated at 100 ng/ml for 12 hr. Cells were fixed and stained with Lamp1 (red). Bar=50 μm. Numbers of GFP-galectin-3 (green) co-localized with Lamp1 per cell were counted and analyzed with one-way ANOVA with Tukey-Kramer method. N=34-57. Data are presented as mean ± S.E., *p<0.05, **p<0.01. Immunofluorescence studies were performed twice.

Supplementary Figure 4

ER stress-induced-lysosomal membrane permeabilization in HK2 cells demonstrated by galectin puncta assay.

HK2 cells expressing pGFP-galectin-3 were cultured with DMSO or 1 μg/ml tunicamycin (TM) or 1 μM thapsigargin (TG) for 12 hr. rhVaspin was co-administrated at 100 ng/ml for 12 hr. Cells were fixed, permeabilized and stained with Lamp1 (red). Bar=50 μm. Number of GFP-galectin-3 (green) colocalized with Lamp1 per cell were counted and analyzed with Bonferroni method. N=15-25. Data are presented as mean ± S.E., *p<0.05, **p<0.01. Immunofluorescence studies were performed twice.

Supplementary Figure 5

Western blot analysis of HK2 cells treated with shRNA-CTRL or shRNA-GRP78.

One μg/ml tunicamycin (TM)- or 1 μM thapsigargin (TG)-induced up-regulation of p62, ATF4

and CHOP were suppressed by rhVaspin administration in shRNA-CTRL HK2 cells, while such beneficial effects of rhVaspin were disappeared in shRNA-GRP78 HK2 cells. Incubation time of TM, TG with rhVaspin was 24 hr. Densitometry quantification of western blot shown in lower panel. White bar (☐); shRNA-CTRL, Gray bar (■); shRNA-GRP78. N=3-6. Statistical analyses are performed in following groups; group 1 (DMSO, TM, DMSO+rhVaspin, and TM+rhVapsin) and group 2 (DMSO, TG, DMSO+rhVaspin, and TG+rhVaspin). The identical data of DMSO or DMSO+rhVaspin are used both in groups 1 and 2, because the analyzed samples were run on the same membrane in upper panels. One-way ANOVA with Tukey-Kramer method is used for multiple comparisons in GRP78/αTubulin, p62/αTubulin in TM of shRNA-GRP78, ATF4/αTubulin in TG of shRNA-CTRL, CHOP/αTubulin in TM of shRNA-GRP78 and TG. Benfferoni method is used for multiple comparisons in p62/αTubulin in TM of shRNA-CTRL and TG, ATF4/αTubulin in TM and TG of shRNA-GRP78, and CHOP/αTubulin in TM of shRNA-CTRL. Data are presented as mean ± S.E., *p<0.05, **p<0.01. Western blots were performed twice.

Supplementary Figure 6

Expression of HSPA1L of vaspin interactive molecule in kidney.

a. Immunoprecipitation of rhVaspin protein-injected mouse kidney using vaspin antibody (upper panel) and using HSPA1L antibody (lower panel). Forming complex of vaspin and HSPA1L is observed *in vivo*. **b**. *Hspa1l* gene expression in C57BL/6J mouse kidney. **c**. HSPA1L expression of HK2 cells cultured with 40 mM mannitol as an osmotic control. **d.** Immunohistochemical staining of HSPA1L using kidney tissues from Vaspin Tg, WT and Vaspin-/- mice. **e.** Quantifications of p62 and HSPA1L in HK2 and H4-II-E-C3 cells, those representative images are shown in **Figure 6h, i and j**. N=3-5. The multiple comparisons are performed by one-way ANOVA with Tukey-Kramer method and two-pair comparisons by Student's *t* test. Data are presented as mean ± S.E., *p<0.05, **p<0.01. Western blots and immunoperoxidase experiments were performed twice.

Supplementary Figure 7

Immunoprecipitation using HEK293T cells expressing p3xFLAG-GFP-HSPA1L with ANTI-FLAG M2 affinity agarose gel. Complex formation of HSPA1L and Lamp2 is observed. Western blots were performed twice.

Supplementary Figure 8

Complex formations of HSPA1L/clathrin heavy chain (CHC) and GRP78/CHC, and subsequent facilitation of endocytosis of rhVaspin.

a. **and b**. HK2 cells expressing p3xFLAG-GFP-HSPA1L or p3xFLAG-GRP78 were cultured with rhVaspin for 24 hr. Cell lysates were used for the detection of rhVaspin. Overexpression of HSPA1L or GRP78 increased cellular rhVaspin protein levels. Densitometry quantification of Western blot is shown on the right of western blot images. N=3-4, Unpaired 2-tailed *t* test is used. **c**. Co-culture of rhVaspin with anti-GRP78 or anti-HSPA1L antibody inhibited rhVaspin protein levels in HK2 cell cultured for 24 hr. HK2 lacks intrinsic expression of vaspin and the internalization of rhVaspin into HK2 cells is inhibited by both antibodies. Densitometry quantification of Western blot is shown in the lower panels. N=3, Unpaired 2-tailed *t* test is used. **d**. HK2 cells expressing pMyc-CHC were cultured with rhVaspin for 24 hr. rhVaspin levels of cell lysate increased in a Myc-CHC dose-dependent manner. Densitometry quantification of Western blot is shown in right panel. N=3, one-way ANOVA followed by Tukey-Kramer method is used. All Data are presented as mean ± S.E., $*p<0.05$, $**p<0.01$. **e**. HEK293T cells expressing p3xFLAG or p3xFLAG-GFP-HSPA1L and pMyc-CHC were immunoprecipitated with ANTI-FLAG M2 affinity agarose gel. Western blot shows a complex formation of HSPA1L and CHC. **f**. HEK293T cells expressing p3xFLAG or p3xFLAG-GRP78 and pMyc-CHC were immunoprecipitated with ANTI-FLAG M2 affinity agarose gel. Complex formation of GRP78 and CHC is demonstrated. Western blots were performed twice.

Supplementary Figure 9

Immunofluorescence staining of Lamp2 (green) and DAPI (blue) in human renal biopsy samples. DN; diabetic nephropathy, TBMD; thin basement membrane disease, ORKD; obesity-related kidney disease, u-pro (g/gCr); urinary protein (g/g•Creatinine). Enlarged lysosome is indicated by arrows. Bar=10 μm. Immunofluorecence studies were performed twice.

Supplementary Figure 10

Immunofluorescence staining of HSPA1L (green) and AQP1 (red) in human renal biopsy samples. IgA; IgA nephropathy, DN; diabetic nephropathy, TBMD; thin basement membrane disease, MGA; minor glomerular abnormality, g; glomerulus, d; distal tubule, arrow; HSPA1L in distal tubule. Bar=10 μm. Immunofluorecence studies were performed twice.

Supplementary Figure 11

Immunofluorescence staining of GRP78 (red) and AQP1 (green) in human renal biopsy samples. IgA; IgA nephropathy, ORKD; obesity-related kidney disease, DN; diabetic nephropathy, TBMD; thin basement membrane disease, MGA; minor glomerular abnormality. Bar=10 μm.

Supplementary Figure 12

Proposed mechanism of vaspin protecting proximal tubular cells. Vaspin protects proximal tubular cells (PTCs) coordinating with GRP78 and HSPA1L, through ameliorating excessive ER stress, autophagy failure, lysosomal membrane permeabilization, NLRP3 inflammasome activation and cell death. Albumin-induced depletion of HSPA1L of PTCs along with increased HSPA1L secretion is a novel mechanism of albuminuria-induced disturbance in homeostasis of PTCs.

Supplementary Figure 13
Uncropped images of the blots included in the figures.

Supplementary Figure 1

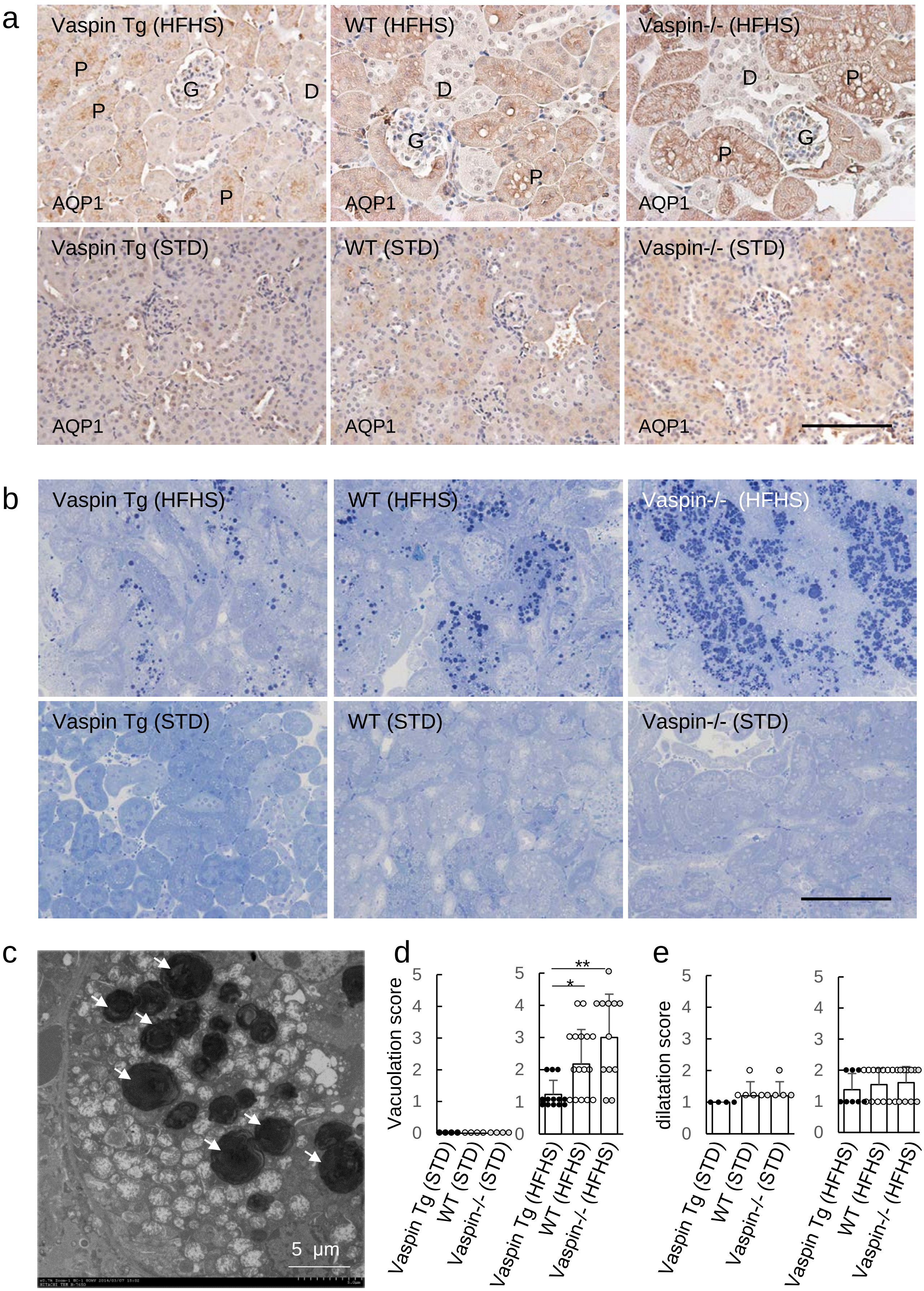

Supplementary Figure 1, continued

f

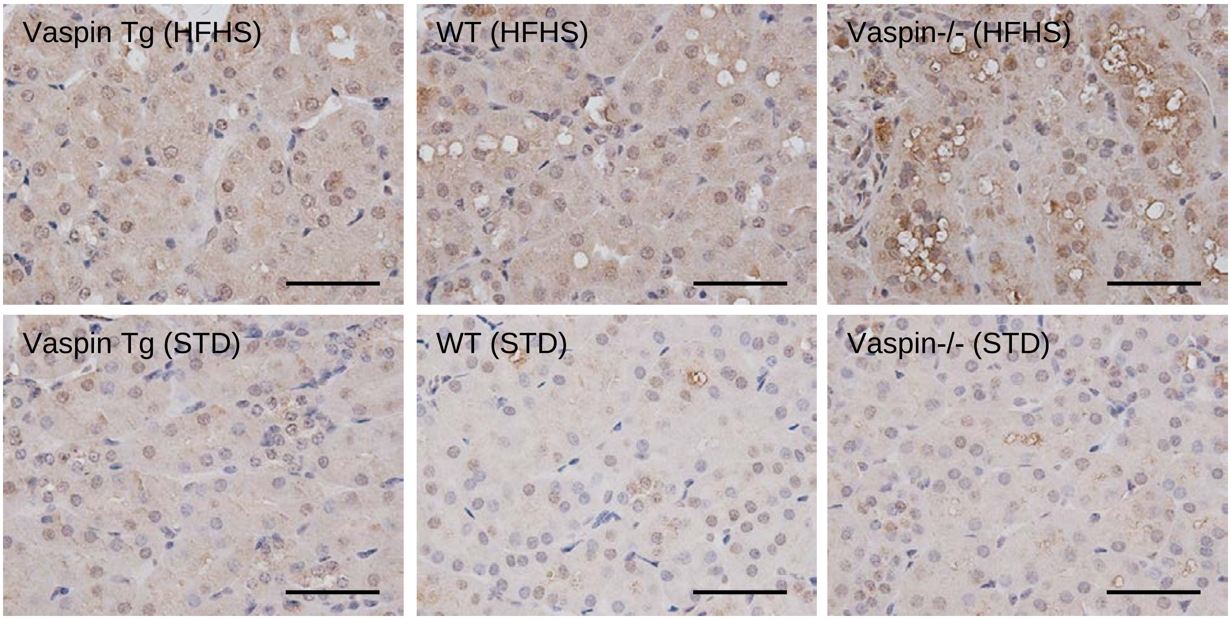

g

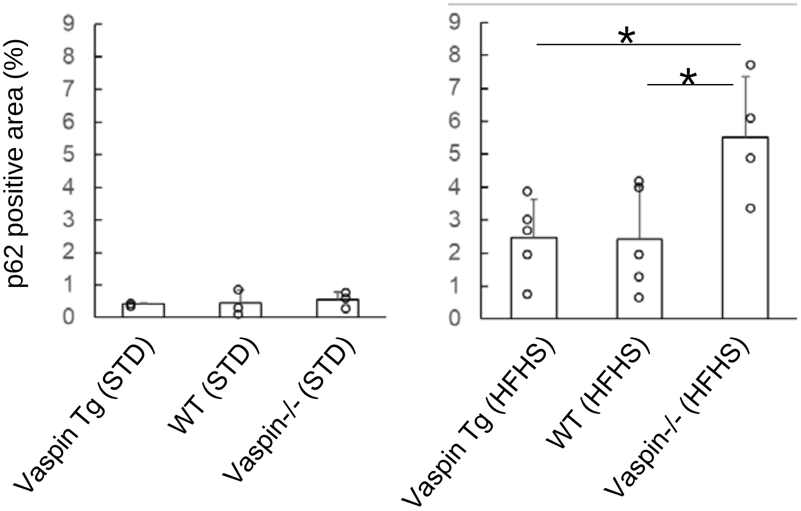

Supplementary Figure 1, continued

h

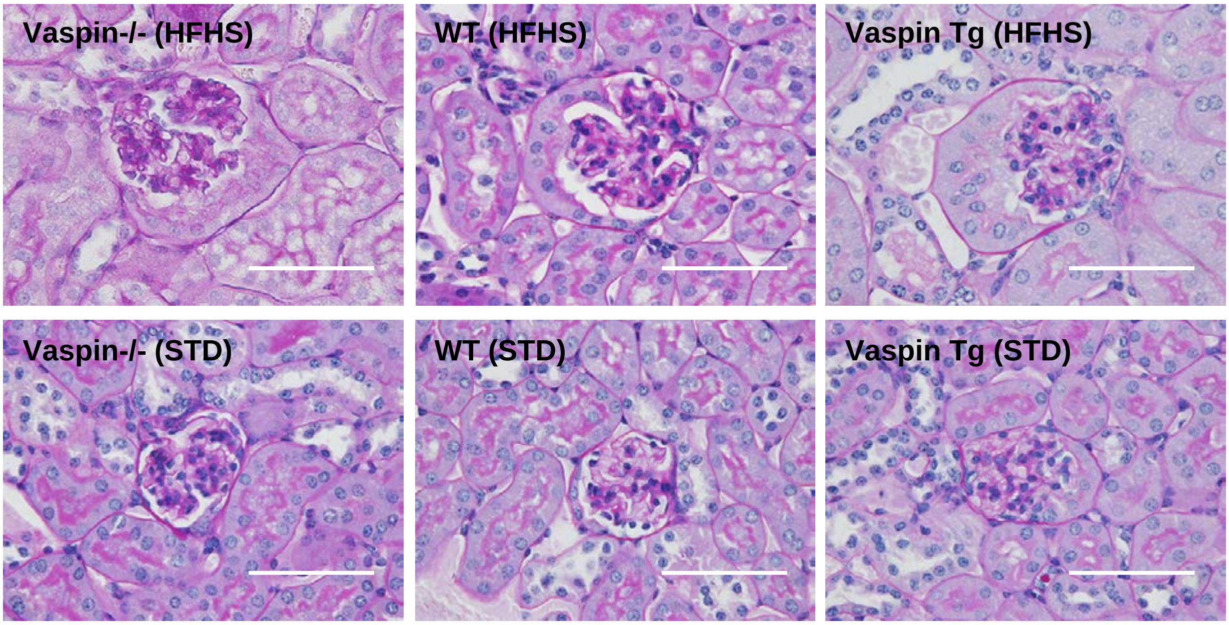

i

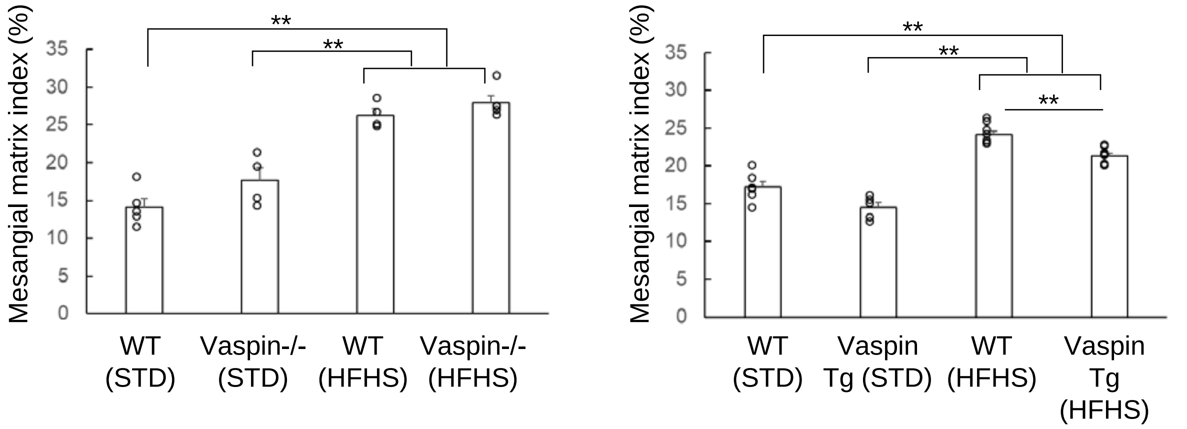

j

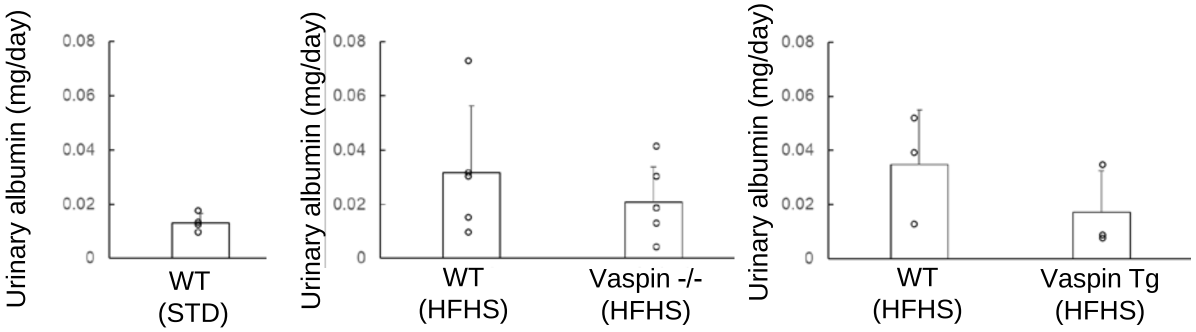

Supplementary Figure 2

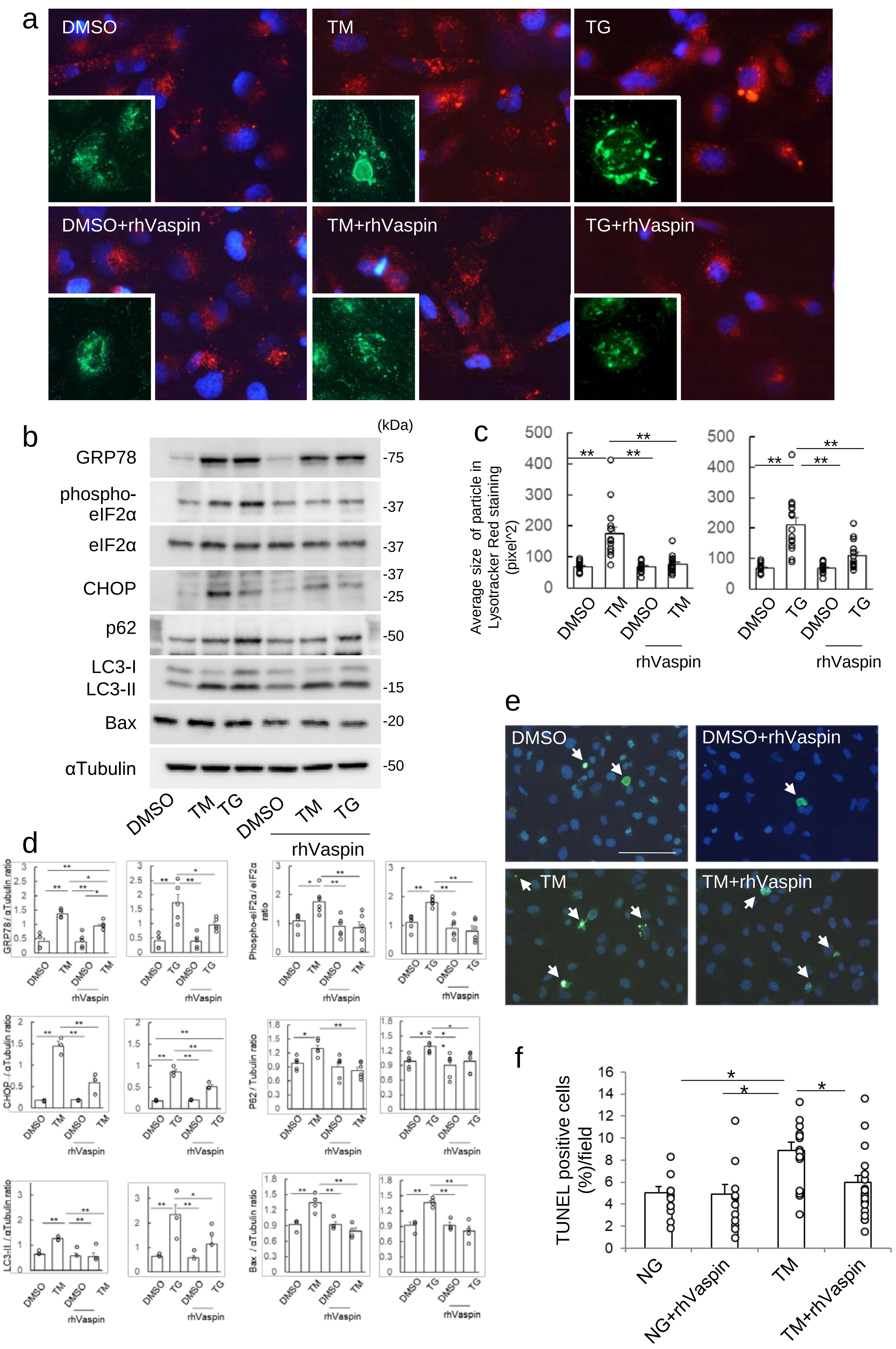

Supplementary Figure 3

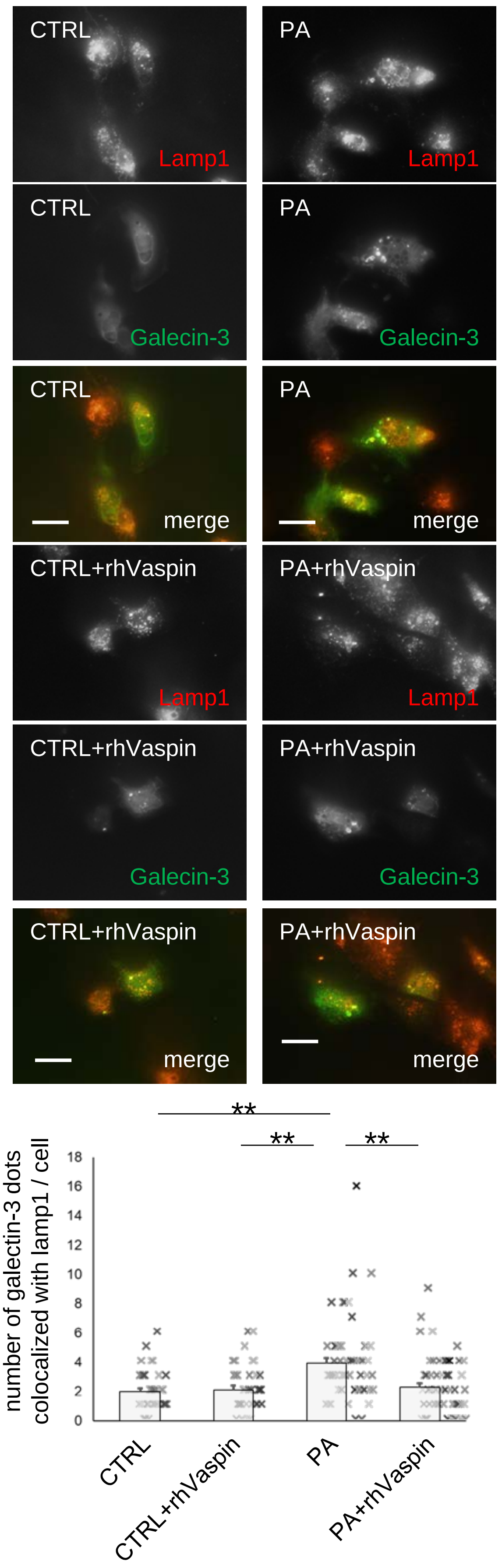

Supplementary Figure 4

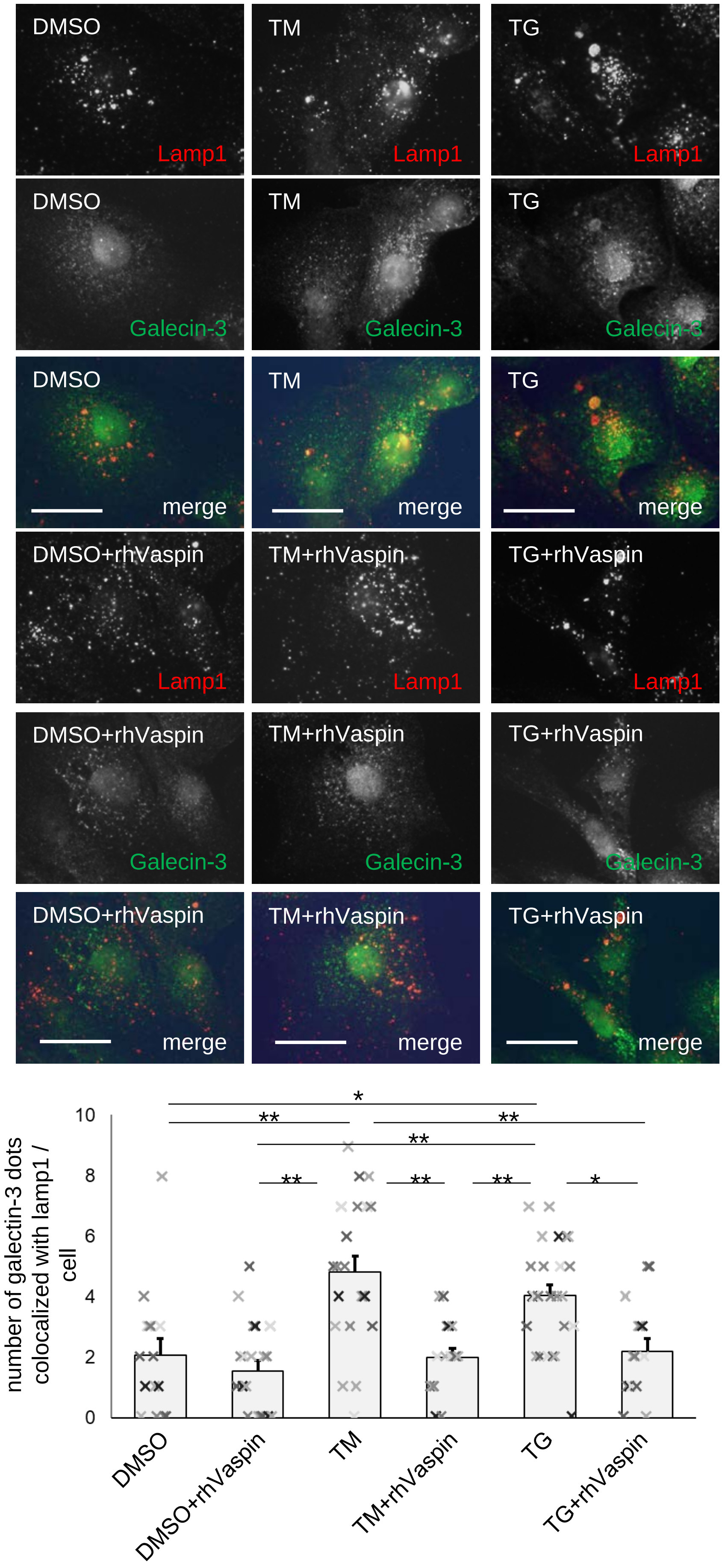

Supplementary Figure 5

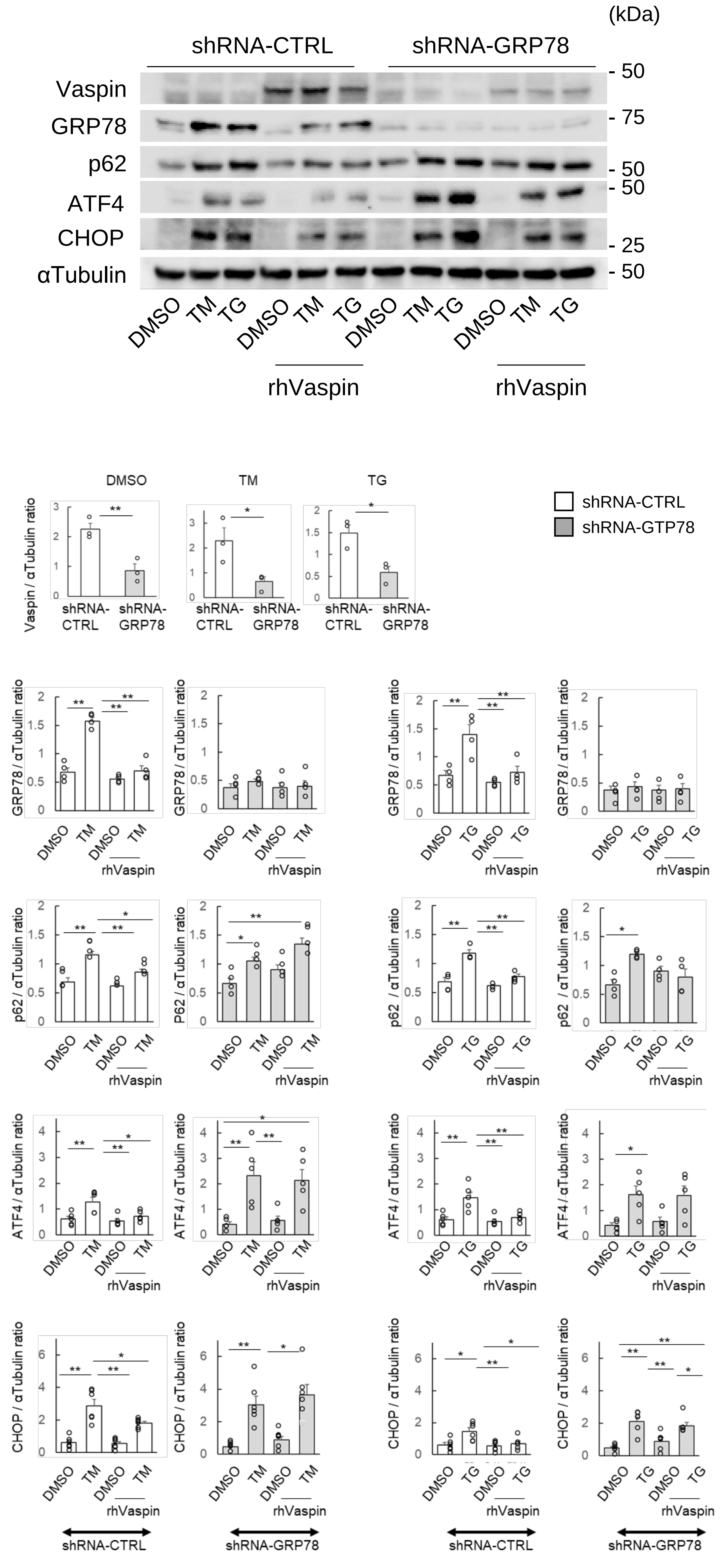

Supplemental Figure 6

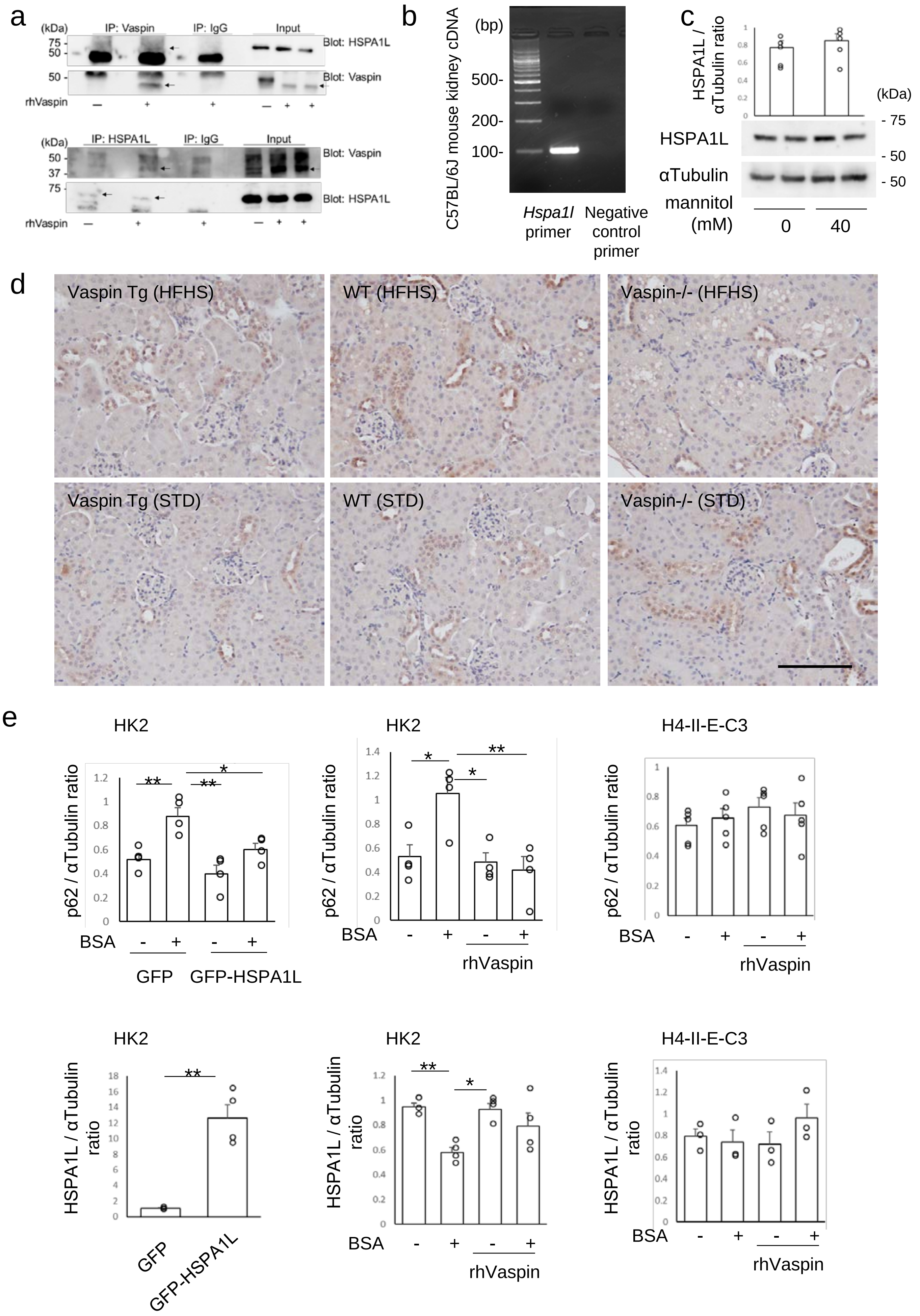

Supplementary Figure 7

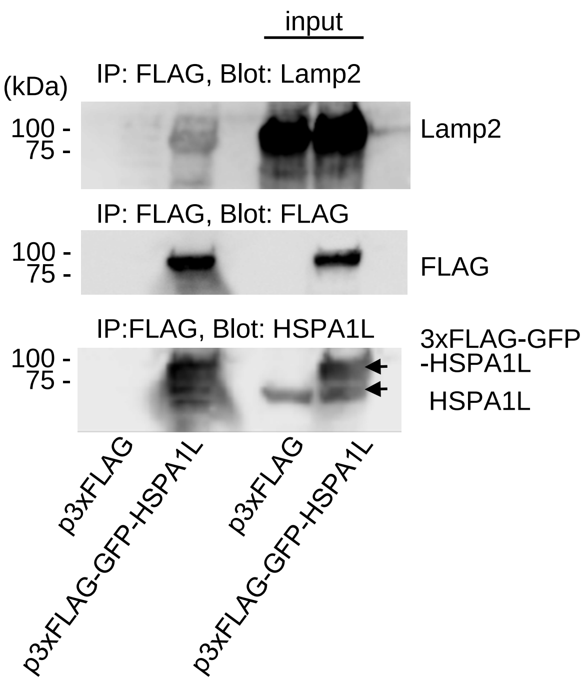

Supplementary Figure 8

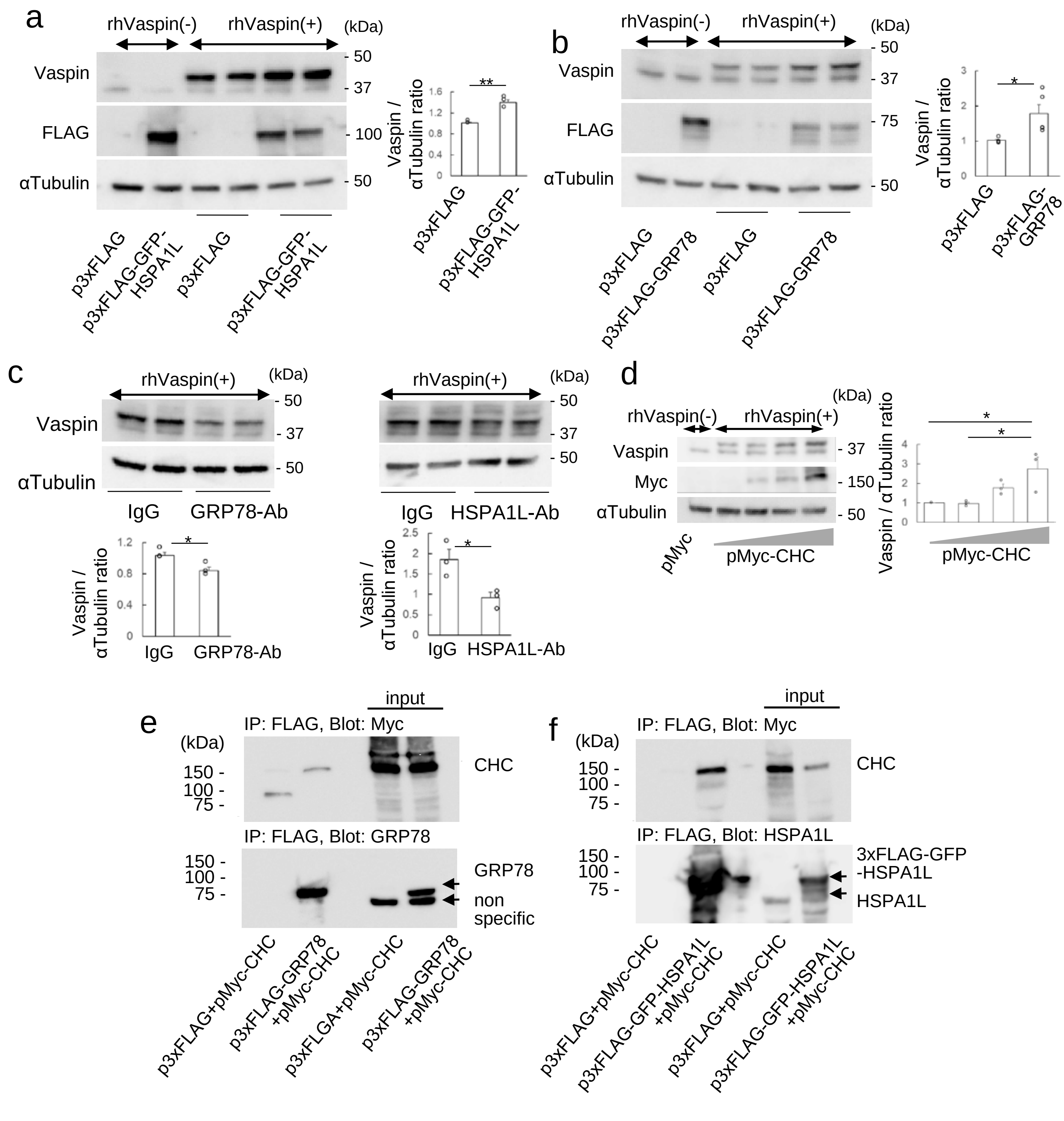

Supplementary Figure 9

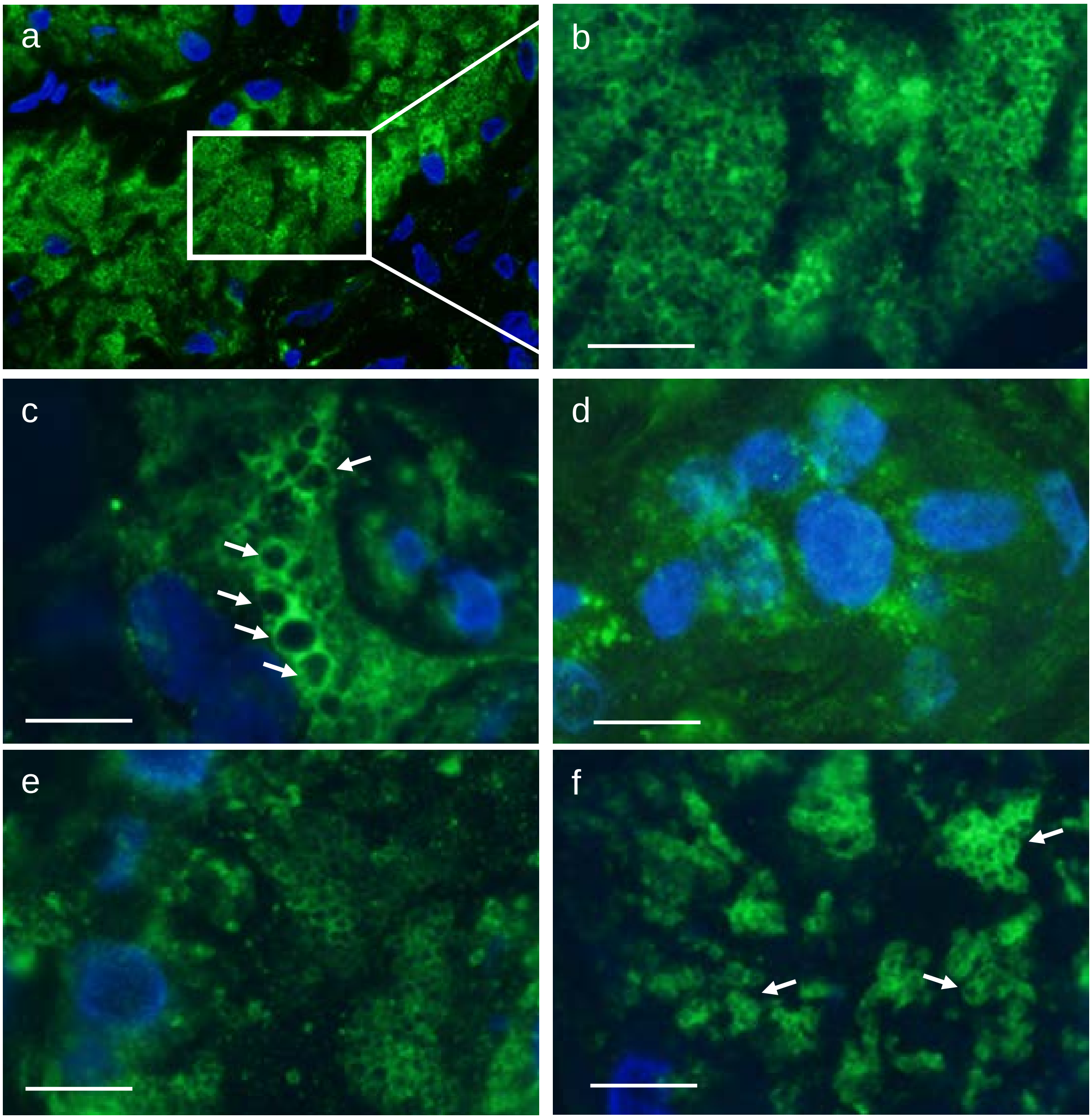

	a, b	c	d	e	f
Age (year)	67	45	40	50	49
M/F	F	F	F	M	M
BMI (kg/m^2)	25.8	27.6	22.6	26.7	28.7
HbA1c (%)	7.4	7.4	5.0	5.5	5.9
u-pro (g/gCr)	3.94	4.5	0.11	0.41	2.16
Diagnosis	DN	DN	TBMD	TBMD	ORKD

DN; diabetic nephropathy, TBMD; thin basement membrane disease, ORKD; obesity-related kidney disease

Supplementary Figure 10

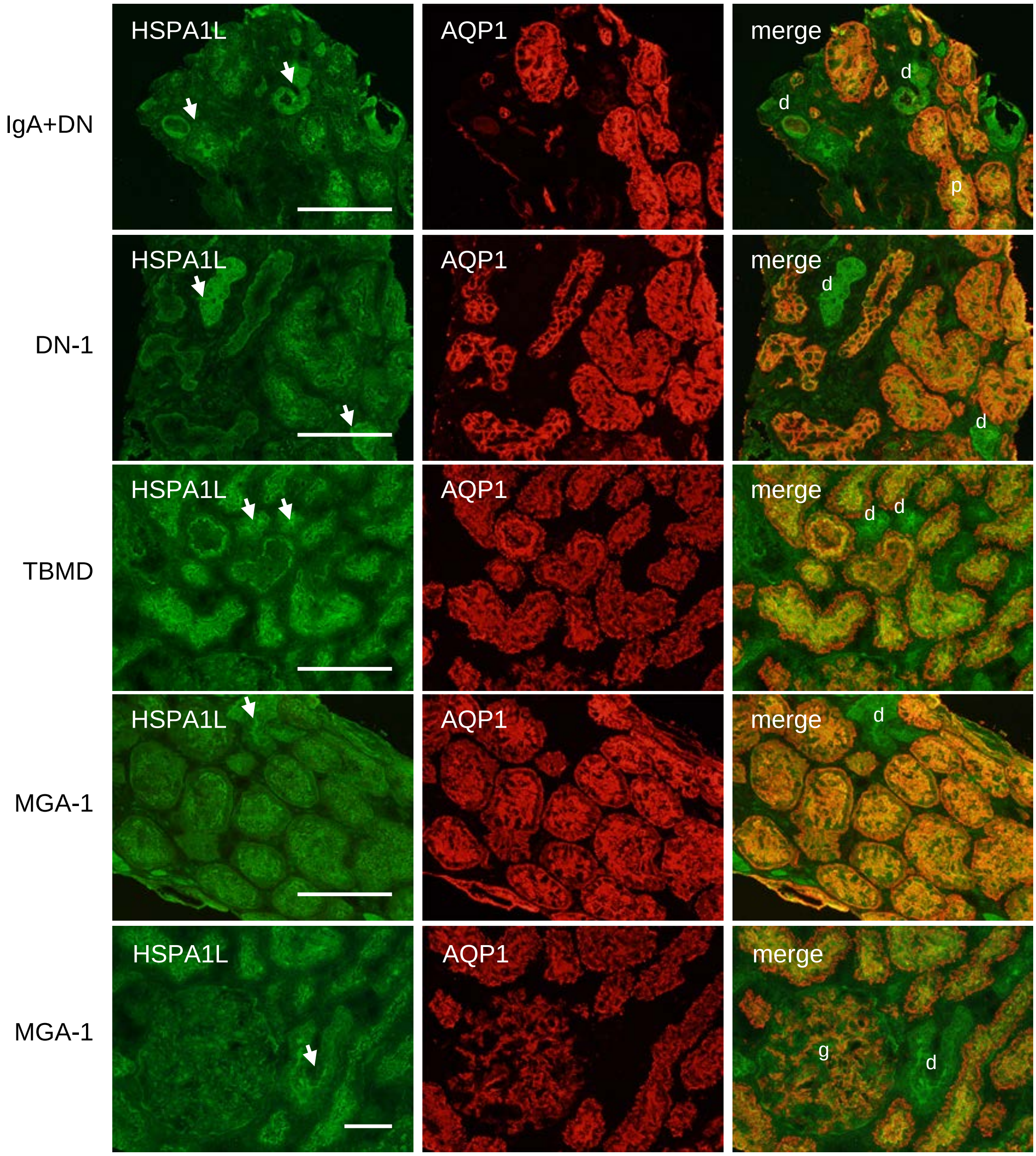

	IgAN+DN	DN-1	TBMD	MGA-1
Age (year)	78	56	40	26
M/F	M	M	F	M
BMI (kg/m^2)	24.4	25.4	22.6	30.7
HbA1c (%)	n.d.	6.5	5.0	5.7
u-pro (g/gCr)	7.56	3.85	0.11	0.37
diagnosis	IgAN,DN	DN	TBMD	MGA

Supplementary Figure 11

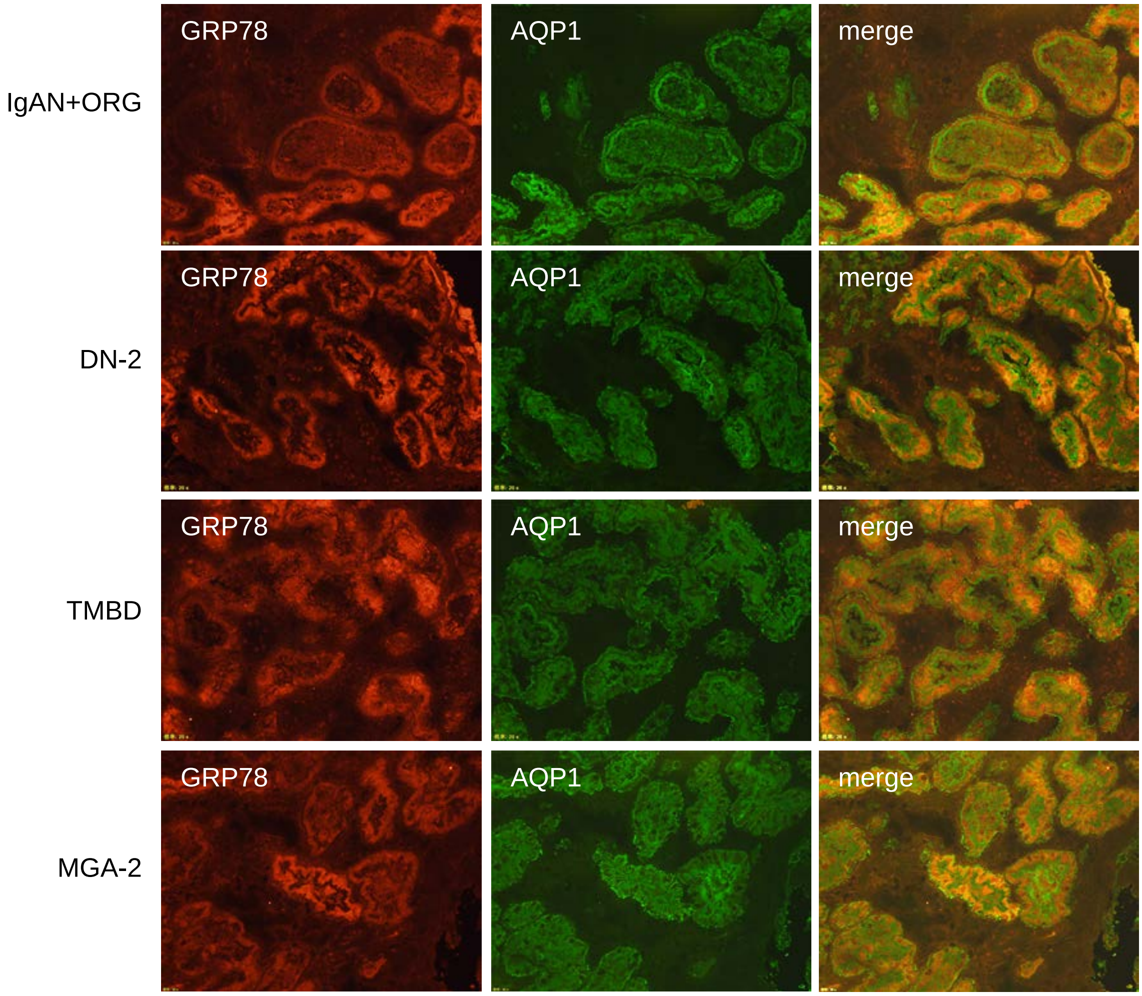

	IgAN+ORG	DN-2	TBMD	MGA-2
Age (year)	30	50	40	41
M/F	M	F	F	M
BMI (kg/m^2)	29.8	31.6	22.6	24.9
HbA1c (%)	8.4	13.8	5.0	5.6
u-pro (g/gCr)	9.52	1.76	0.11	1.39
Diagnosis	IgAN, ORG	DN	TBMD	MGA

Supplementary Figure 12

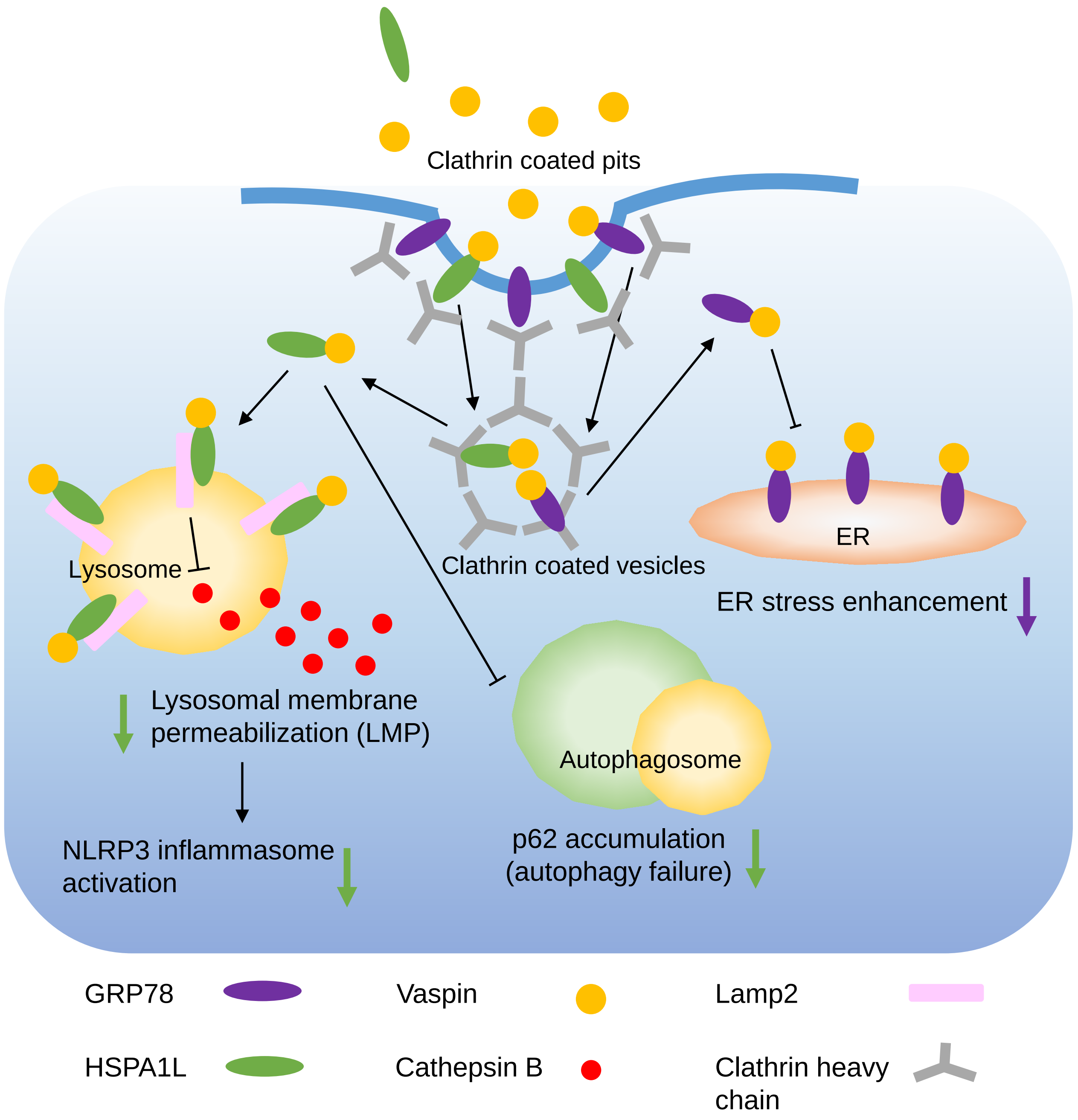

Supplementary Figure 13

Uncropped images in Figure 2

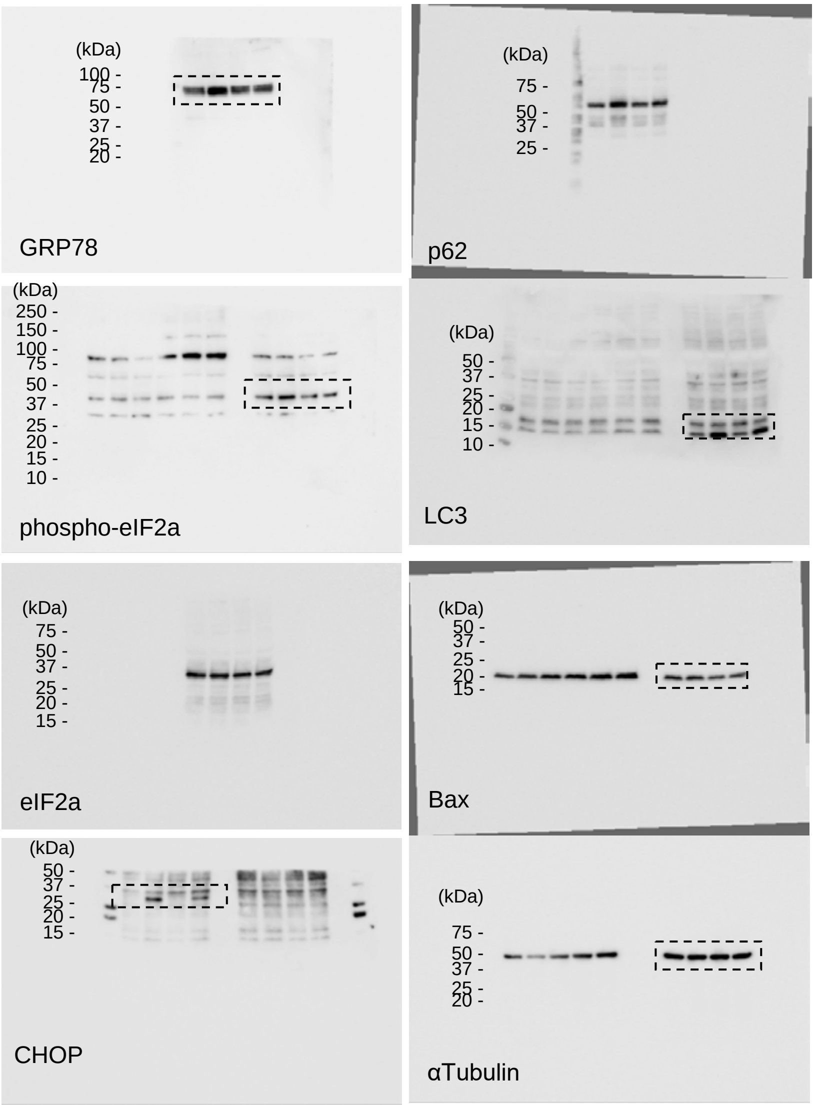

Uncropped images in Figure 3

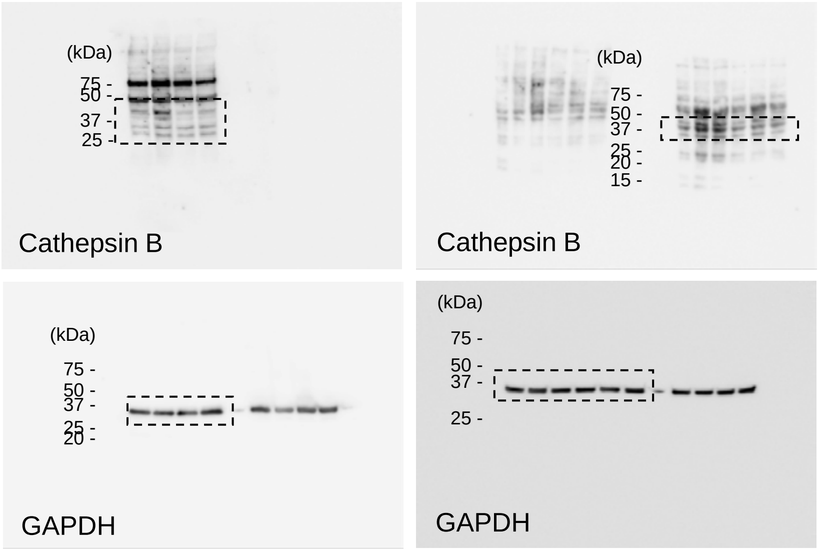

Supplementary Figure 13, continued

Uncropped images in Figure 4

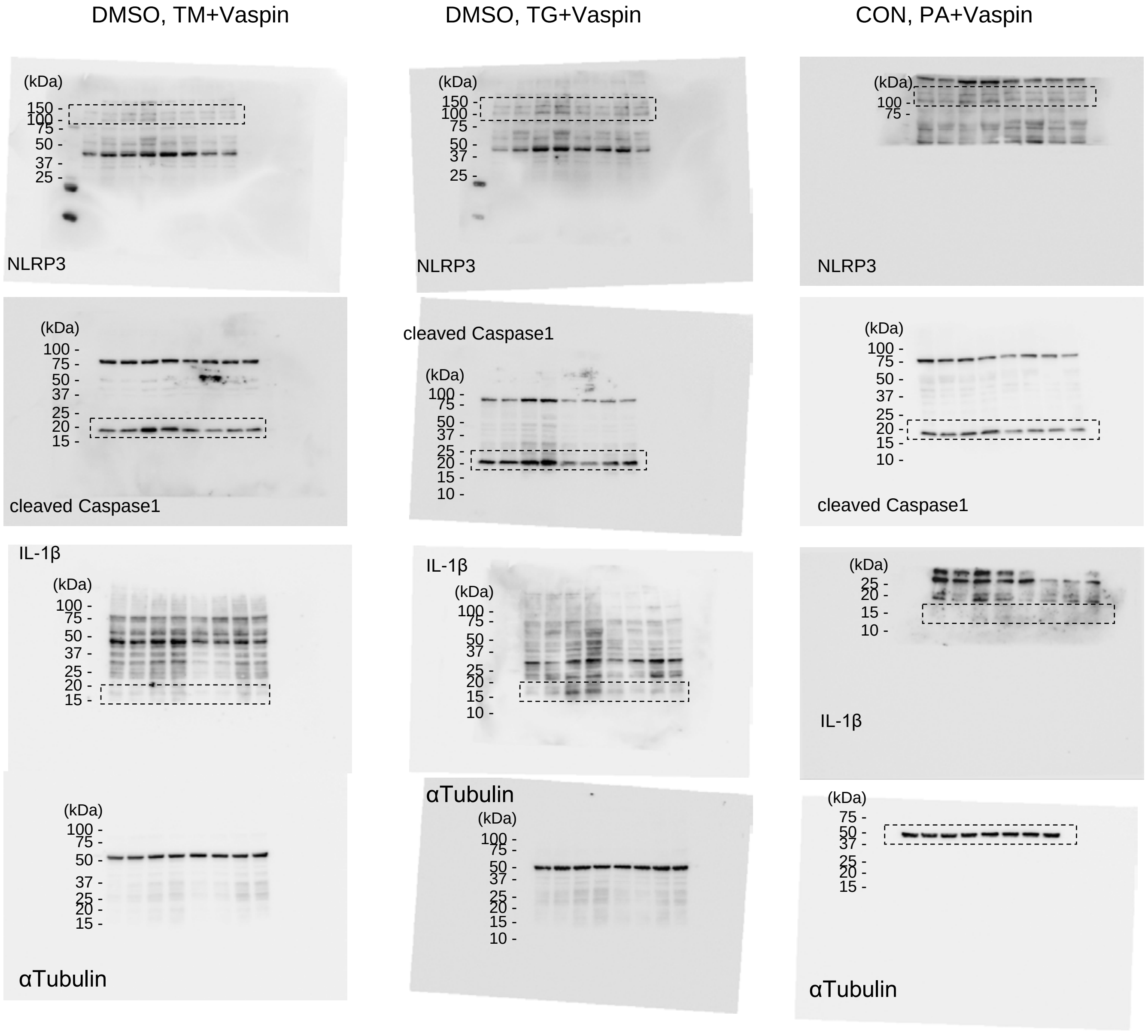

Supplementary Figure 13, continued

Uncropped images in Figure 5

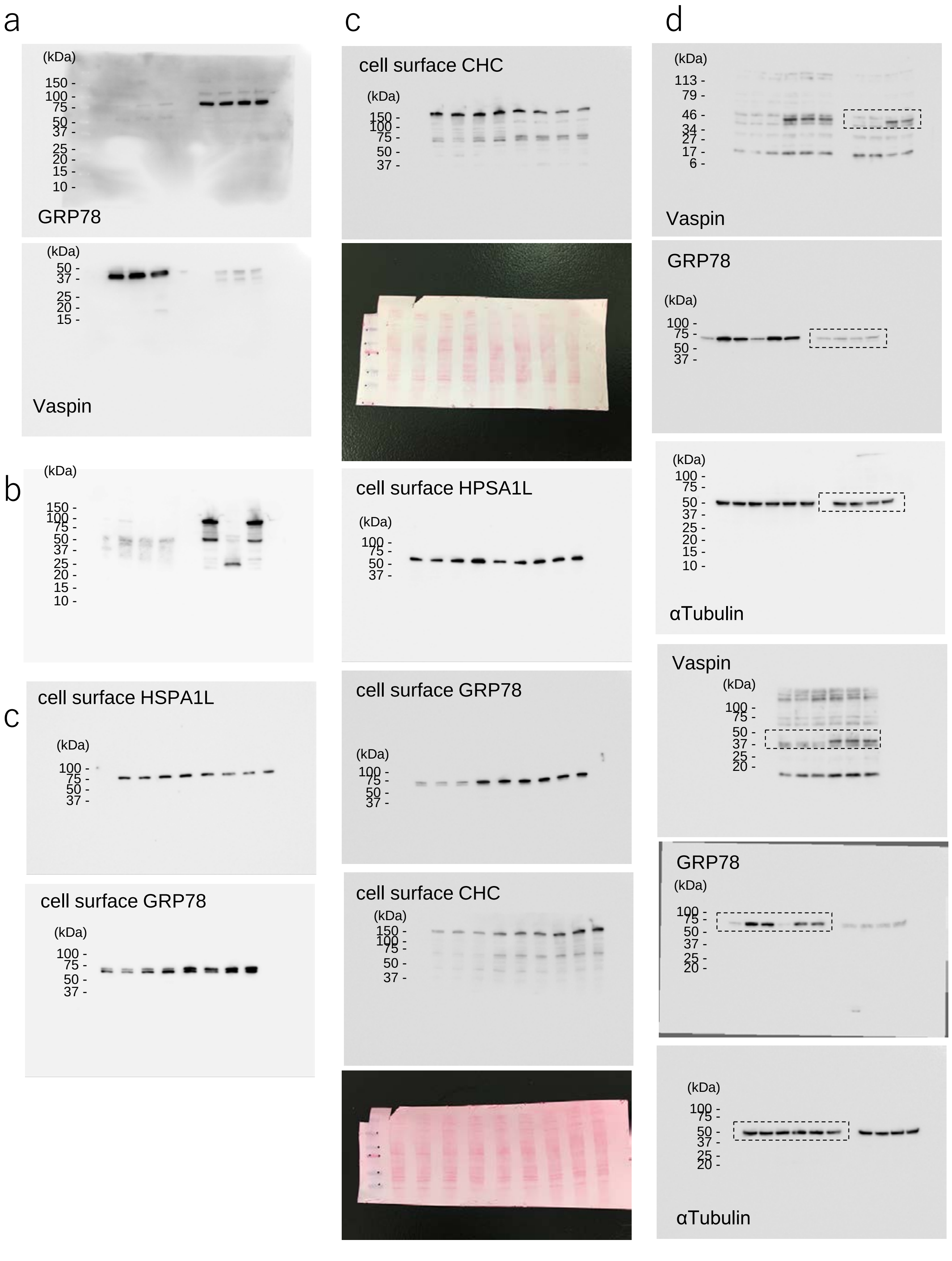

Supplementary Figure 13, continued

Uncropped images in Figure 6

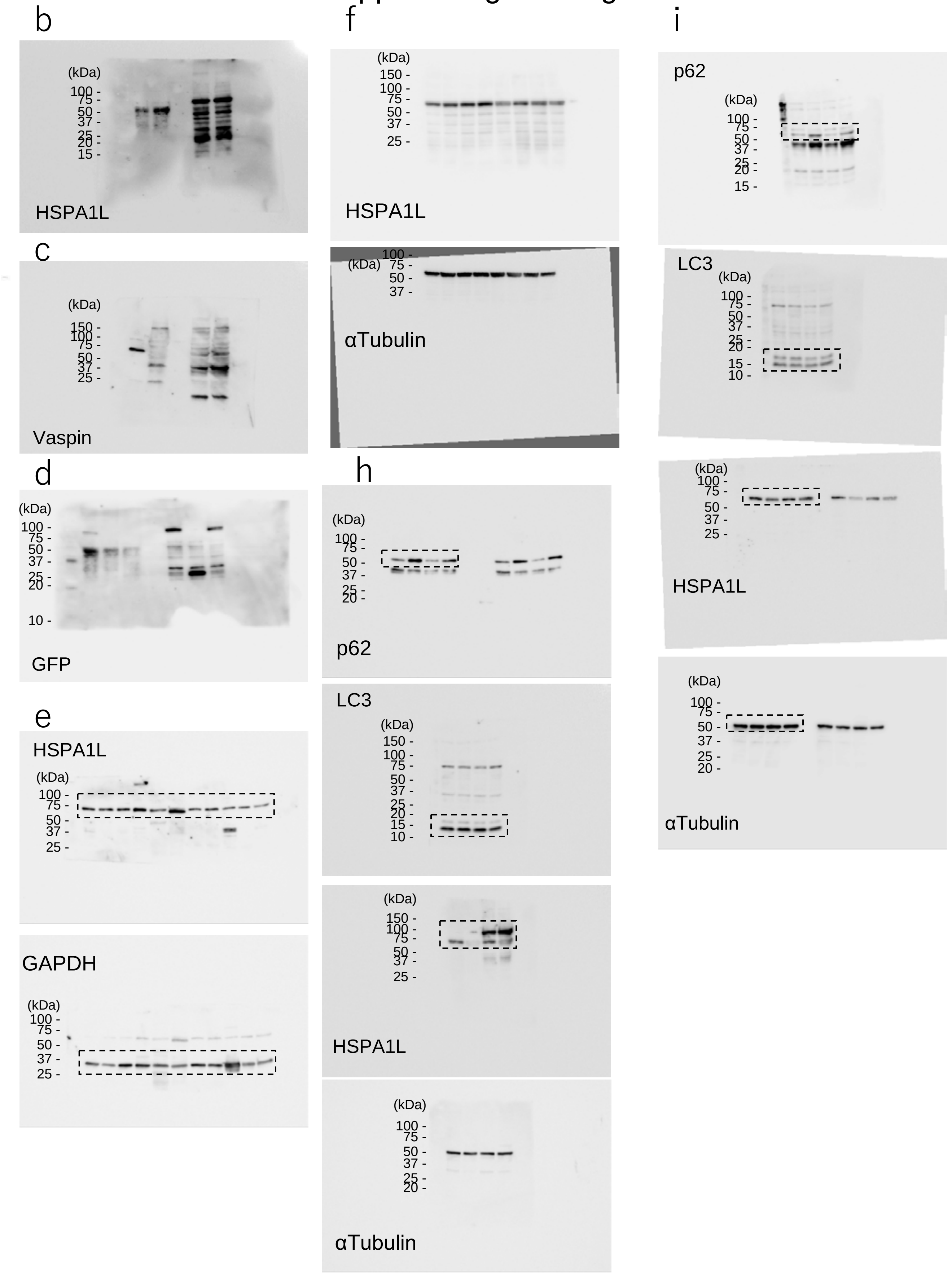

Supplementary Figure 13, continued

Uncropped images in Figure 6

Uncropped images in Figure 7

j

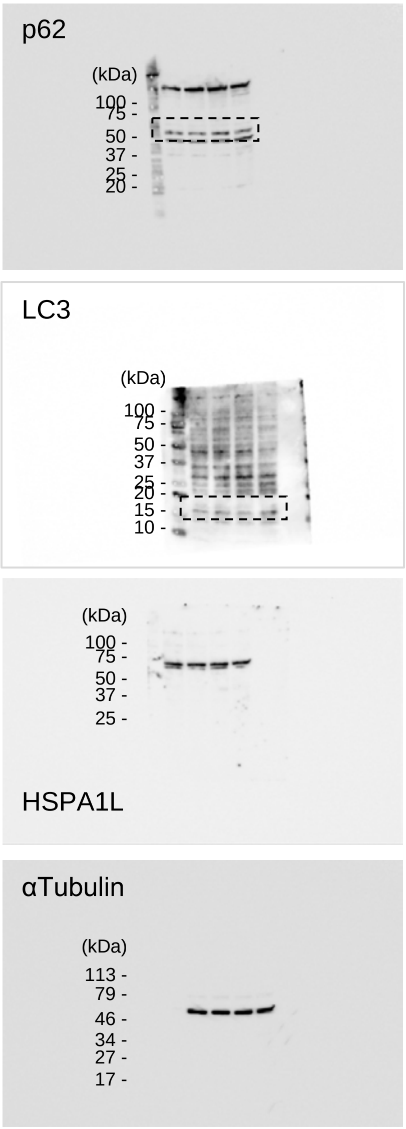

a

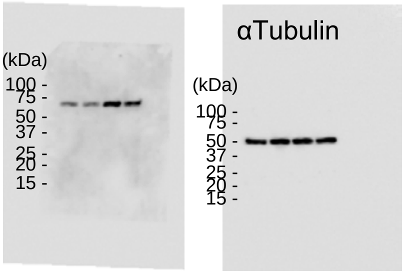

b

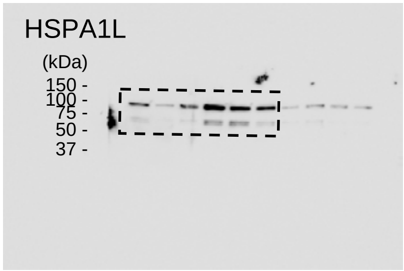

c

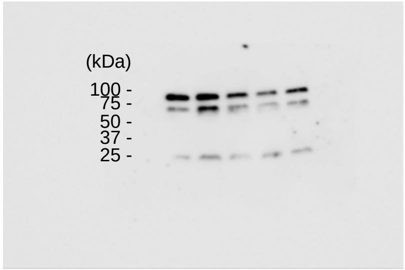

d

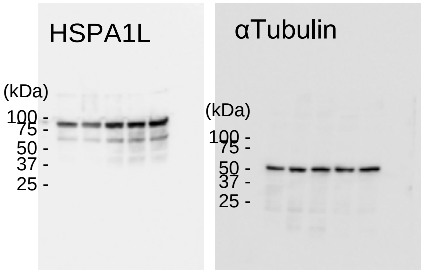

e

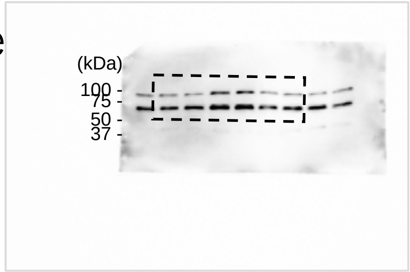

f

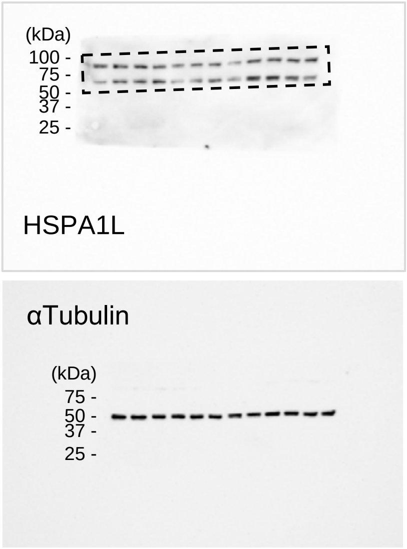

Supplementary Figure 13, continued

Uncropped images in Figure 9

Vaspin-/- mice injected with rhVaspin

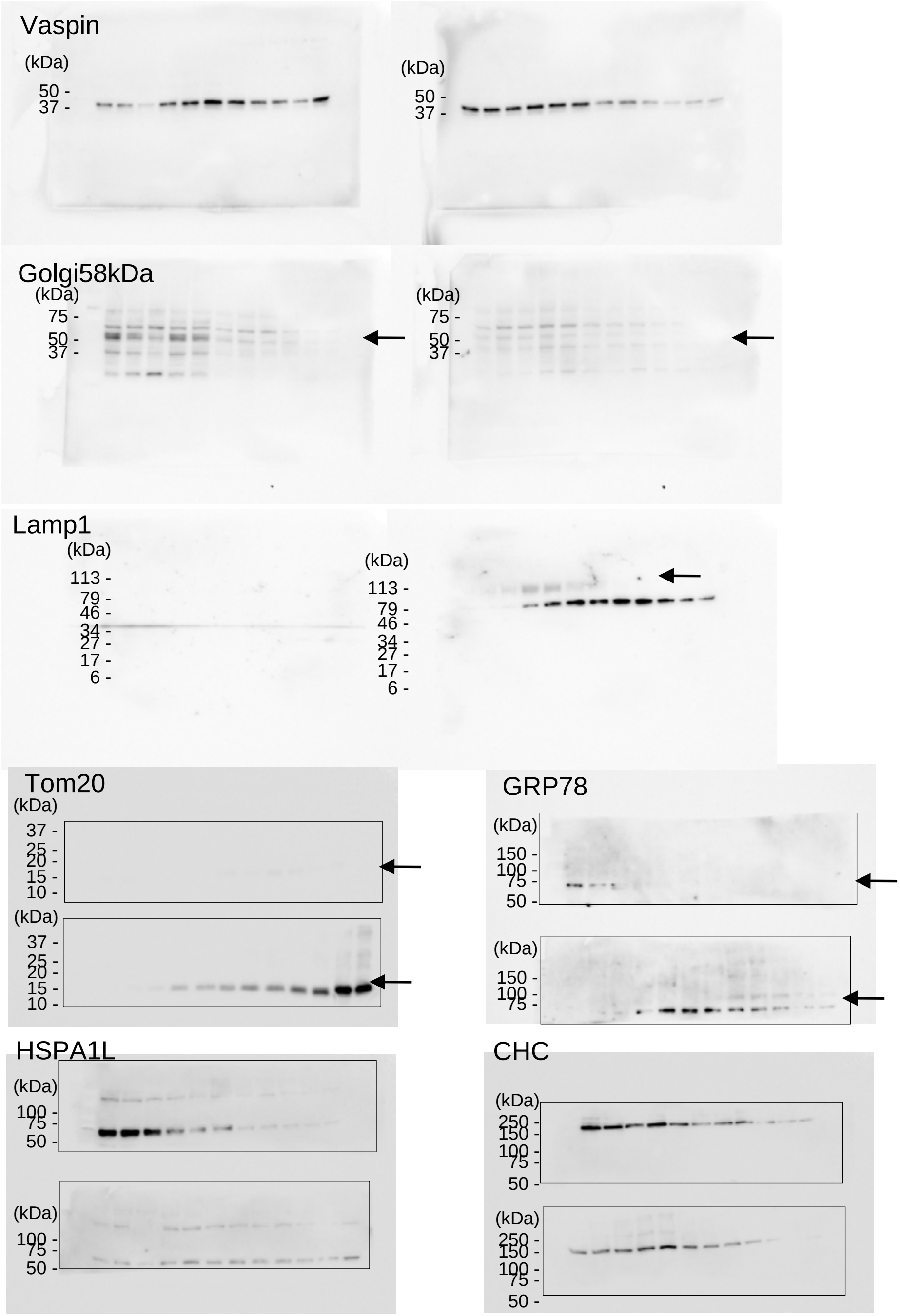

Vaspin-/- mice injected with PBS

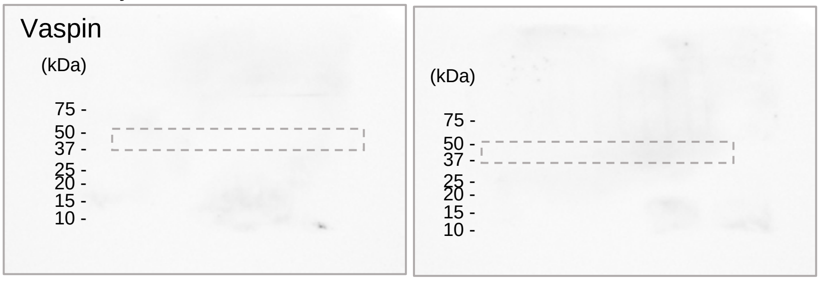

Supplementary Figure 13, continued

Uncropped images in Supplementary Figure 2

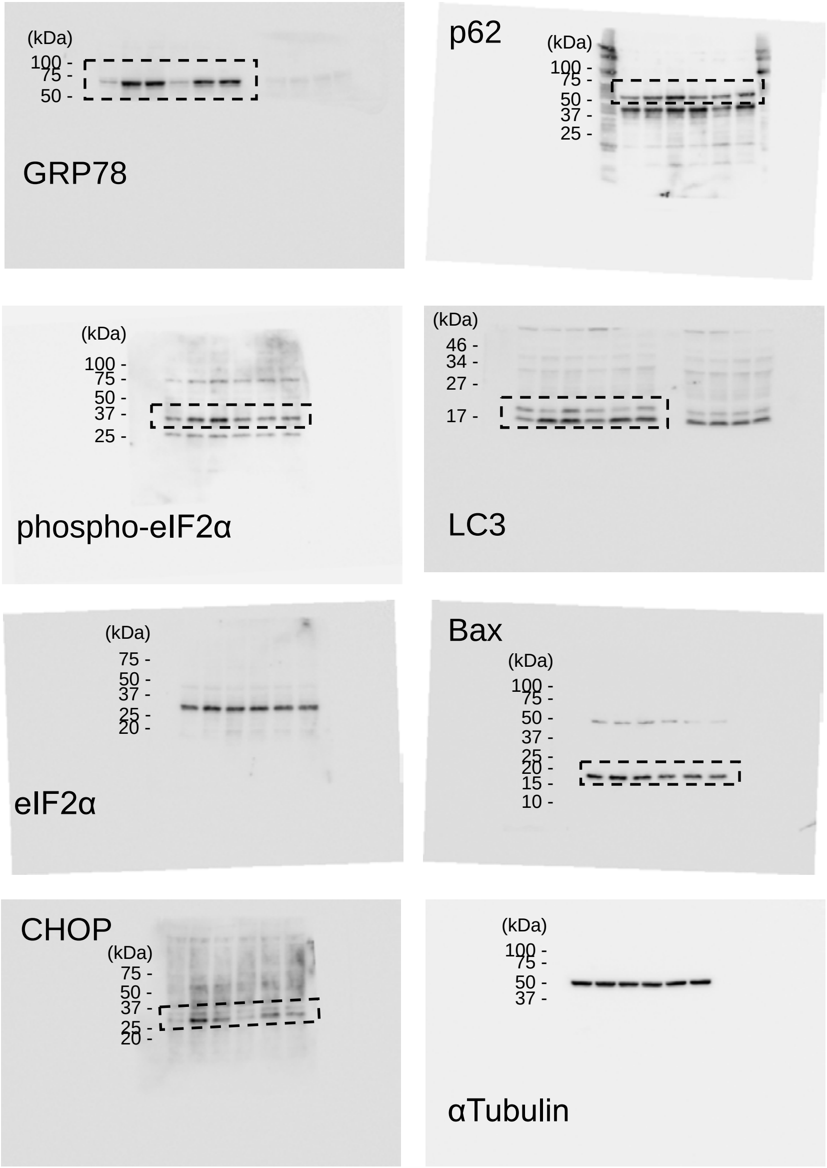

Supplementary Figure 13, continued

Uncropped images in Supplementary Figure 5

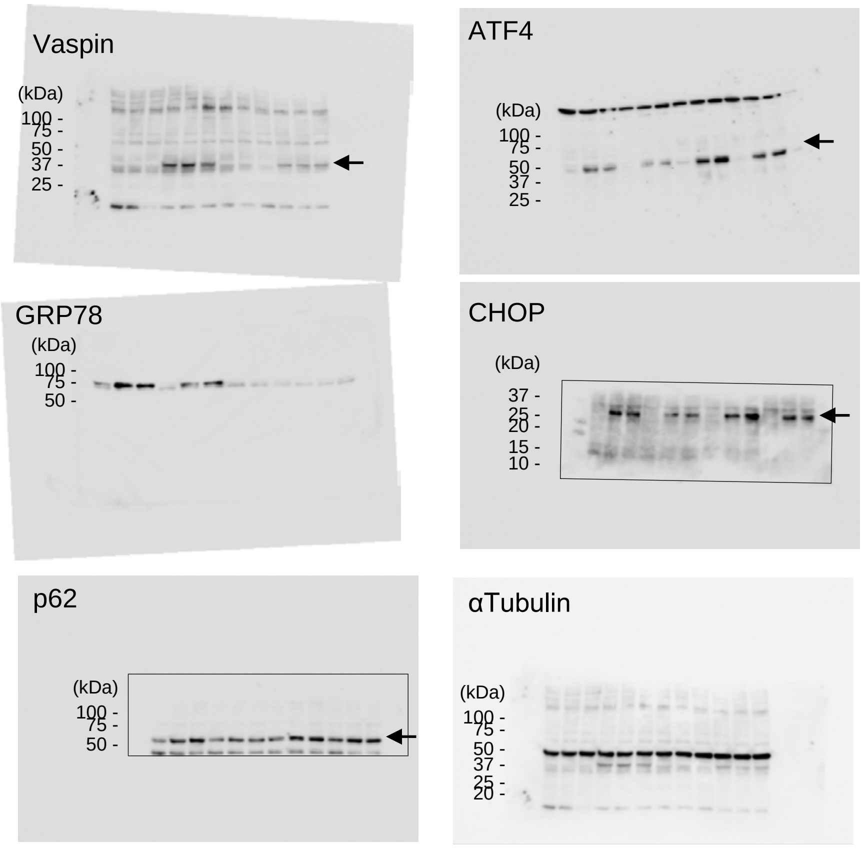

Supplementary Figure 13, continued

Uncropped images in Supplementary Figure 6

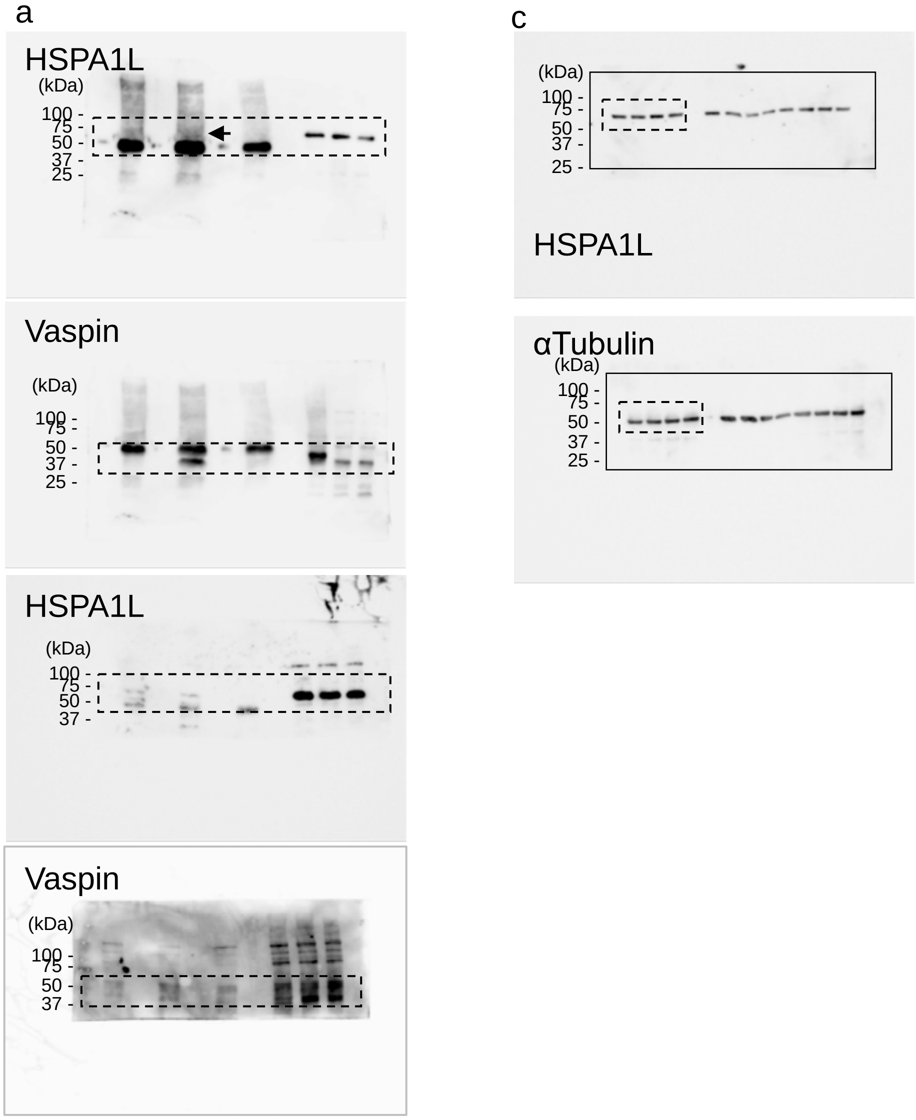

Uncropped images in Supplementary Figure 7

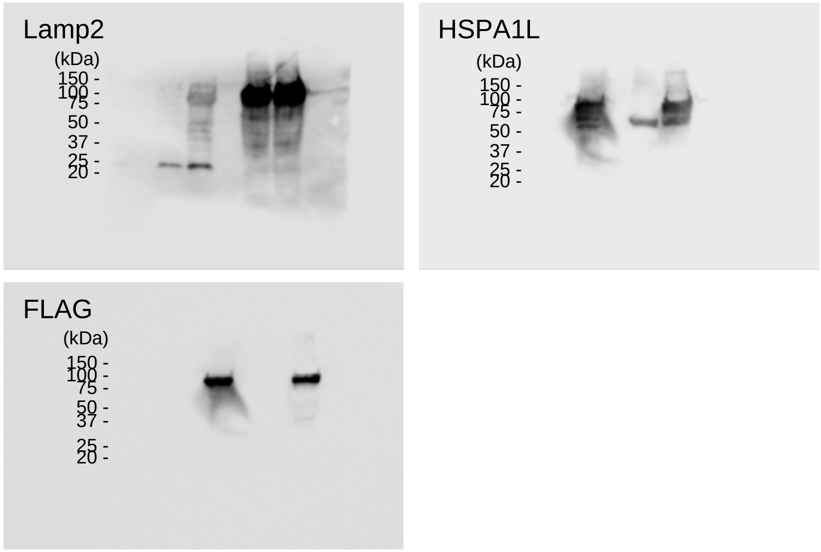

Supplementary Figure 13, continued

Uncropped images in Supplementary Figure 8

a & b

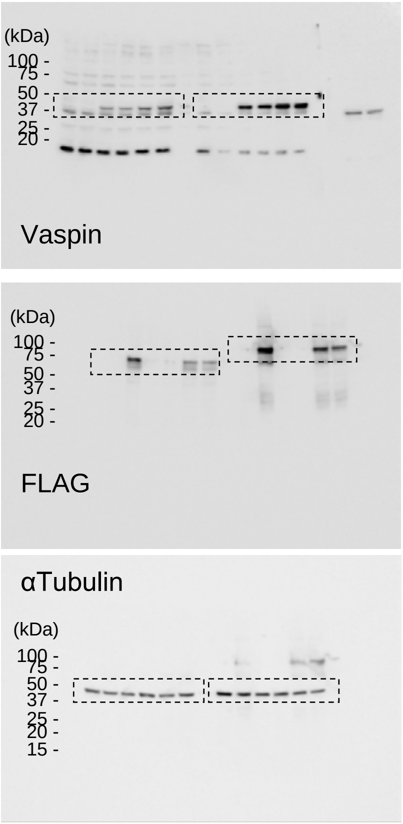

d

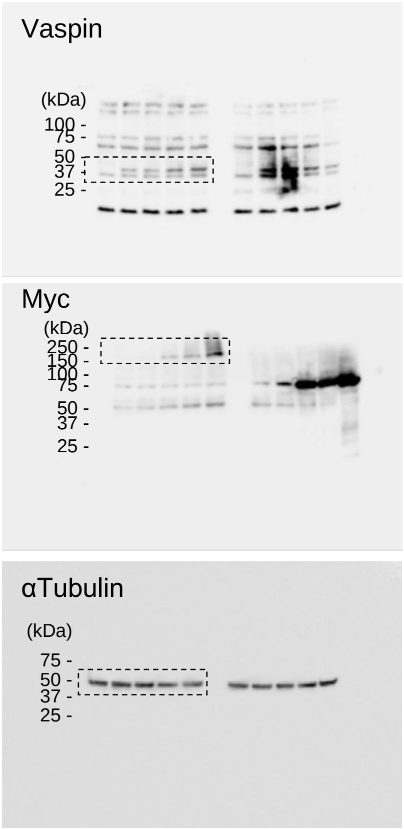

f

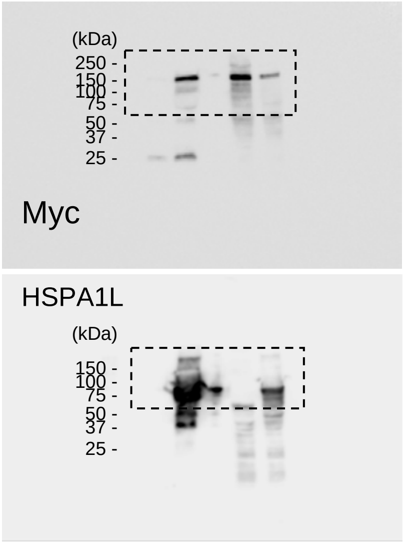

c

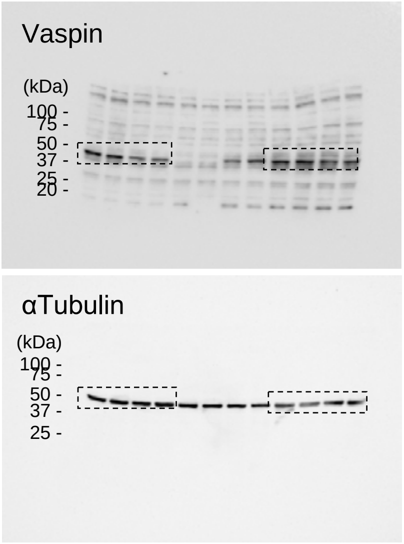

e

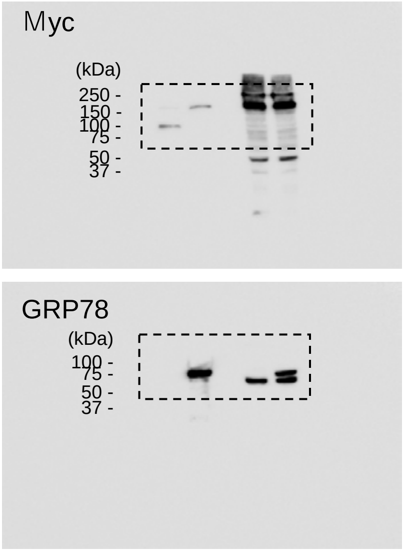

Supplement: Supplementary file 2 — Supplementary Information [file 42003_2021_1902_MOESM2_ESM.pdf]
