# Supplementary material for: A Vaspin–HSPA1L complex protects proximal tubular cells from organelle stress in diabetic kidney disease
Source: Commun Biol. 2021 Mar 19;4:373. doi: 10.1038/s42003-021-01902-y (PMC7979793; doi:10.1038/s42003-021-01902-y)
Supplement: Supplementary file 3 — Description of Additional Supplementary Files [file 42003_2021_1902_MOESM3_ESM.pdf]

Descriptions for Additional Supplementary Files

File Name: Supplementary Data 1 Gene ontology of identified proteins by LC-MS/MS and analysis with Mascot Ver. 2.5.1 and Scaffold Ver. 4.8.3.

Description: Analysis information and setting of Mascot Ver. 2.5.1.

HK-2-1_Swissprot(human), Samples report created on 08/05/2020

Experiment: HK-2-1_Swissprot(human)

- Peak List Generator: unknown
  - Version: unknown
  - Charge States Calculated: unknown
  - Deisotoped: unknown
  - Textual Annotation: unknown
- Database Set: 1 Database
  - Database Name: the SwissProt_2015_04 database
    - Version: unknown
    - Taxonomy: Homo sapiens
    - Number of Proteins: 20205
  - Does database contain common contaminants?: unknown
- Search Engine Set: 1 Search Engine
  - Search Engine: Mascot
    - Version: 2.5.1
    - Samples: All Samples
    - Fragment Tolerance: 0.60 Da (Monoisotopic)
    - Parent Tolerance: 5.0 PPM (Monoisotopic)

Fixed Modifications:

Variable Modifications: +16 on M (Oxidation), +71 on C (Propionamide)

Database: the SwissProt_2015_04 database (selected for Homo sapiens, unknown version, 20205 entries)

Digestion Enzyme: Trypsin

Max Missed Cleavages: 1

Probability Model:

11_HK_2_1 (F046019): Peptide Prophet with Delta Mass Correction (No decoys found) [all charge states]

Scaffold: Version: Scaffold_4.8.3

Modification Metadata Set: 1541 modifications

Source: C:\Program Files\Scaffold 4\parameters\unimod.xml

Comment:

Protein Grouping Strategy: Experiment-wide grouping with protein cluster analysis

Peptide Thresholds: 95.0% minimum and

Protein Thresholds: 1 peptide minimum

Peptide FDR: 0.8% (Prophet)

Protein FDR: 0.9% (Prophet)

GO Annotation Source(s): NCBI (downloaded 2015/05/27)

Alternate ID Source(s):

File Name: Supplementary Data 2

Description: All source data underlying the graphs presented in the main figures.
