# Supplementary material for: A Vaspin–HSPA1L complex protects proximal tubular cells from organelle stress in diabetic kidney disease
Source: Commun Biol. 2021 Mar 19;4:373. doi: 10.1038/s42003-021-01902-y (PMC7979793; doi:10.1038/s42003-021-01902-y)
Supplement: Supplementary file 6 — Reporting Summary [file 42003_2021_1902_MOESM6_ESM.pdf]

nature research

Corresponding author(s): Nakatsuka A and Wada J

Last updated by author(s): Feb 19, 2021

Reporting Summary

Nature Research wishes to improve the reproducibility of the work that we publish. This form provides structure for consistency and transparency in reporting. For further information on Nature Research policies, see our Editorial Policies and the Editorial Policy Checklist.

Statistics

For all statistical analyses, confirm that the following items are present in the figure legend, table legend, main text, or Methods section.

n/a	Confirmed	
☐	☒	The exact sample size (n) for each experimental group/condition, given as a discrete number and unit of measurement
☐	☒	A statement on whether measurements were taken from distinct samples or whether the same sample was measured repeatedly
☐	☒	The statistical test(s) used AND whether they are one- or two-sided<br>*Only common tests should be described solely by name; describe more complex techniques in the Methods section.*
☒	☐	A description of all covariates tested
☐	☒	A description of any assumptions or corrections, such as tests of normality and adjustment for multiple comparisons
☐	☒	A full description of the statistical parameters including central tendency (e.g. means) or other basic estimates (e.g. regression coefficient) AND variation (e.g. standard deviation) or associated estimates of uncertainty (e.g. confidence intervals)
☐	☒	For null hypothesis testing, the test statistic (e.g. F, t, r) with confidence intervals, effect sizes, degrees of freedom and P value noted<br>*Give P values as exact values whenever suitable.*
☒	☐	For Bayesian analysis, information on the choice of priors and Markov chain Monte Carlo settings
☒	☐	For hierarchical and complex designs, identification of the appropriate level for tests and full reporting of outcomes
☒	☐	Estimates of effect sizes (e.g. Cohen's d, Pearson's r), indicating how they were calculated

Our web collection on statistics for biologists contains articles on many of the points above.

Software and code

Policy information about availability of computer code

Data collection	N/A
Data analysis	IBM SPSS Modeler 18.0 was used for statistical analysis. Densitometry of western blot was measured by Image J software.

For manuscripts utilizing custom algorithms or software that are central to the research but not yet described in published literature, software must be made available to editors and reviewers. We strongly encourage code deposition in a community repository (e.g. GitHub). See the Nature Research guidelines for submitting code & software for further information.

Data

Policy information about availability of data

All manuscripts must include a data availability statement. This statement should provide the following information, where applicable:

- Accession codes, unique identifiers, or web links for publicly available datasets
- A list of figures that have associated raw data
- A description of any restrictions on data availability

All data supporting this paper are available in the manuscript or Supplementay information. Raw data are available from authors upon request.

Field-specific reporting

Please select the one below that is the best fit for your research. If you are not sure, read the appropriate sections before making your selection.

☒ Life sciences ☐ Behavioural & social sciences ☐ Ecological, evolutionary & environmental sciences

For a reference copy of the document with all sections, see nature.com/documents/nr-reporting-summary-flat.pdf

Life sciences study design

All studies must disclose on these points even when the disclosure is negative.

Sample size	No statistical methods were used to predetermine sample size, in vivo and in vitro analysis.
Data exclusions	No data were excluded from the analysis.
Replication	All experiments were repeated at least one more experiment.
Randomization	The samples for in vivo and in vitro were randomly allocated into experimental groups.
Blinding	The investigators were blinded to group allocation during data collection and/or analysis.

Reporting for specific materials, systems and methods

We require information from authors about some types of materials, experimental systems and methods used in many studies. Here, indicate whether each material, system or method listed is relevant to your study. If you are not sure if a list item applies to your research, read the appropriate section before selecting a response.

Materials & experimental systems

n/a	Involved in the study	
☐	☒	Antibodies
☐	☒	Eukaryotic cell lines
☒	☐	Palaeontology and archaeology
☐	☒	Animals and other organisms
☐	☒	Human research participants
☐	☒	Clinical data
☒	☐	Dual use research of concern

Methods

n/a	Involved in the study	
☒	☐	ChIP-seq
☒	☐	Flow cytometry
☒	☐	MRI-based neuroimaging

Antibodies

Antibodies used

The following antibodies were used for western blot analysis.
anti-BiP (C50B12) Rabbit mAb (catalog number: #3177, Cell Signaling Technology)
anti-phospho-eIF2α (Ser51) (D9G8) XP® Rabbit mAb (catalog number: #3398, Cell Signaling Technology)
anti-ATF-4 (D4B8) Rabbit mAb (catalog number: #11815, Cell Signaling Technology)
anti-GAPDH (14C10) Rabbit mAb (catalog number: #2118, Cell Signaling Technology)
anti-eIF2α (catalog number: #9722, Cell Signaling Technology)
anti-Bax (catalog number: #2772, Cell Signaling Technology)
anti-CHOP (L63F7) Mouse mAb (catalog number: #2895, Cell Signaling Technology)
anti-αTubulin (DM1A) Mouse mAb (catalog number: #3873, Cell Signaling Technology)
anti-Myc-Tag (9B11) Mouse mAb (catalog number: #2276, Cell Signaling Technology)
Anti-Cathepsin B antibody [CA10] (catalog number: ab58802, Abcam)
Anti-LAMP2 antibody [H4B4], (catalog number: ab25631, Abcam)
Anti-NLRP3 antibody (catalog number: ab4207, ab214185, Abcam),
Anti-Caspase 1 antibody (catalog number: ab17820, Abcam)
Rabbit polyclonal to Clathrin heavy chain (catalog number: ab21679, Abcam)
Anti-SERPINA12 antibody (Biotin) (catalog number: ab58975, Abcam)
Anti-HSPA1L antibody (catalog number: ab154403, Abcam)
Anti-GFP antibody (catalog number: ab290, Abcam)
Anti-LAMP1 antibody (catalog number: ab24170, Abcam)
Anti-IL 1 beta antibody (catalog number: ab9722, Abcam)
Anti-LC3 pAb (catalog number: PM036, MBL)
Anti-p62 (SQSTM1) pAb (catalog number: PM045, MBL)
GRP78 (76-E6) (catalog number: sc-13539, Santa Cruz Biotechnology)
Monoclonal ANTI-FLAG M2, Clone M2 (catalog number: F1804, SIGMA-ALDRICH)
Mouse IgG, HRP-Linked Whole Ab Sheep (catalog number: NA931, GE healthcare Life Sciences)

Rabbit IgG, HRP-Linked Whole Ab Donkey (catalog number: NA934, GE healthcare Life Sciences)
donkey anti-goat IgG-HRP (catalog number: sc-2020, Santa Cruz Biotechnology)
Anti-rat IgG, HRP-linked Antibody (catalog number: #7077, Cell Signaling Technology).
Rabbit TrueBlot, anti-Rabbit IgG HRP (catalog number: 18-8815-33, ROCKLAND)

The following antibodies were used for immunofluorescence microscopy analysis.
Anti-Cathepsin B antibody [CA10] (catalog number: ab58802, Abcam)
Anti-LAMP1 antibody (catalog number: ab24170, Abcam)
Anti-LAMP2 antibody [GL2A7], (catalog number: ab13524, Abcam)
Anti-HSPA1L antibody (catalog number: ab154403, Abcam)
Cathepsin B (D1C7Y) XP?Rabbit mAb (catalog number: #31718, Cell Signaling Technologies)
Anti-p62 (SQSTM1) pAb (catalog number: PM045, MBL)
AQP1 (B-11) (catalog number: sc-25287, Santa Cruz Biotechnology)
BiP (C50B12) Rabbit mAb (catalog number: #3177, Cell Signaling Technologies)
Chicken anti-Mouse IgG (H+L) Cross-Adsorbed Secondary Antibody, Alexa Fluor 488 (catalog number: A-21200, Invitrogen)
Donkey anti-Rat IgG (H+L) Highly Cross-Adsorbed Secondary Antibody, Alexa Fluor 488 (catalog number: A-21208, Invitrogen)
Chicken anti-Rabbit IgG (H+L) Cross-Adsorbed Secondary Antibody, Alexa Fluor 488 (catalog number: A-21441)
Goat anti-Mouse IgG (H+L) Cross-Adsorbed Secondary Antibody, Alexa Fluor 594 (catalog number: A-11005, Invitrogen)
Anti-rabbit IgG (H+L), F(ab')2 Fragment (Alexa Fluor® 555 Conjugate) (catalog number: #4413, Cell Signaling Technologies)

The following antibodies were used for Immunohistochemistry.
Anti-LAMP1 antibody (catalog number: ab24170, Abcam)
Anti-SERPINA12 antibody (Biotin) (catalog number: ab58975, Abcam),
Anti-IL 1 beta antibody (catalog number: ab9722, Abcam)
anti-AQP1 (H-55) (catalog number: sc-20810, Santa Cruz Biotechnology)
Anti-HSPA1L antibody (catalog number: ab154403, Abcam)
Anti-BiP (C50B12) (catalog number: #3177, Cell Signaling Technology)
Goat Anti-Rabbit IgG H&L (Biotin) (catalog number: ab6720, Abcam)
Anti-p62 (SQSTM1) pAb (catalog number: PM045, MBL)

Validation

Validations of the others are shown in data sheets or manufacturer's website.

Eukaryotic cell lines

Policy information about cell lines

Cell line source(s)	HK2 and HEK293T were purchased from ATCC. H4-II-E-C3 were purchased from ECACC.
Authentication	The cell lines were purchased from authorized company, and none of the cell lines used were authenticated afterward.
Mycoplasma contamination	None of the cell lines showed signs of mycoplasma contamination, however we didn't tested it.
Commonly misidentified lines (See ICLAC register)	No commonly misidentified lines were used.

Animals and other organisms

Policy information about studies involving animals; ARRIVE guidelines recommended for reporting animal research

Laboratory animals	Vaspin transgenic C57BL/6J male mice under aP2 promoter and Vaspin knockout (Vaspin-/-) mice (ref.11) were housed under 12-hour light-dark cycle and had free access to water.
Wild animals	This study didn't involve wild animals.
Field-collected samples	Not applicable.
Ethics oversight	The Animal Care and Use Committee of the Department of Animal Resources, Advanced Science Research Center, Okayama University

Note that full information on the approval of the study protocol must also be provided in the manuscript.

Human research participants

Policy information about studies involving human research participants

Population characteristics	Patient who clinically received renal biopsy due to differential diagnosis for proteinuria and/or microhematuria.
Recruitment	Frozen kidney sections obtained from clinical renal biopsies at Okayama University Hospital were used for immunofluorescence analysis. We retrospectively selected the patients with obesity related kidney disease and type 2 diabetic nephropathy.
Ethics oversight	Okayama University Hospital, Ethics Committee

Note that full information on the approval of the study protocol must also be provided in the manuscript.

Clinical data

Policy information about clinical studies

All manuscripts should comply with the ICMJE guidelines for publication of clinical research and a completed CONSORT checklist must be included with all submissions.

Clinical trial registration	n/a
Study protocol	n/a
Data collection	n/a
Outcomes	n/a
